# Obliquity pacing of the western Pacific Intertropical Convergence Zone over the past 282,000 years

**DOI:** 10.1038/ncomms10018

**Published:** 2015-11-25

**Authors:** Yi Liu, Li Lo, Zhengguo Shi, Kuo-Yen Wei, Chien-Ju Chou, Yi-Chi Chen, Chih-Kai Chuang, Chung-Che Wu, Horng-Sheng Mii, Zicheng Peng, Hiroshi Amakawa, George S. Burr, Shih-Yu Lee, Kristine L. DeLong, Henry Elderfield, Chuan-Chou Shen

**Affiliations:** 1 CAS Key Laboratory of Crust-Mantle Material and Environment, School of Earth and Space Science, University of Science and Technology of China, Hefei 230026, China; 2 High-Precision Mass Spectrometry and Environment Change Laboratory (HISPEC), Department of Geosciences, National Taiwan University, No. 1, Sec. 4, Roosevelt Road, Taipei 10617, Taiwan ROC; 3 State Key Laboratory of Loess and Quaternary Geology, Institute of Earth Environment, Chinese Academy of Sciences, Xi'an 710075, China; 4 CAS Center for Excellence in Tibetan Plateau Earth Sciences, Beijing 100101, China; 5 Department of Earth Sciences, National Taiwan Normal University, Taipei 11677, Taiwan ROC; 6 Department of Physics, University of Arizona, Tucson, Arizona 85721, USA; 7 Research Center for Environmental Changes, Academia Sinica, Taipei 11529, Taiwan ROC; 8 Department of Geography and Anthropology, Louisiana State University, Baton Rouge, Louisiana 70803, USA; 9 Department of Earth Sciences, University of Cambridge, Cambridge CB2 3EQ, UK

## Abstract

The Intertropical Convergence Zone (ITCZ) encompasses the heaviest rain belt on the Earth. Few direct long-term records, especially in the Pacific, limit our understanding of long-term natural variability for predicting future ITCZ migration. Here we present a tropical precipitation record from the Southern Hemisphere covering the past 282,000 years, inferred from a marine sedimentary sequence collected off the eastern coast of Papua New Guinea. Unlike the precession paradigm expressed in its East Asian counterpart, our record shows that the western Pacific ITCZ migration was influenced by combined precession and obliquity changes. The obliquity forcing could be primarily delivered by a cross-hemispherical thermal/pressure contrast, resulting from the asymmetric continental configuration between Asia and Australia in a coupled East Asian–Australian circulation system. Our finding suggests that the obliquity forcing may play a more important role in global hydroclimate cycles than previously thought.

## Abstract (web summary)

Predicting future migrations in the Intertropical Convergence Zone—Earth's heaviest rain belt—is limited by a lack of long-term records. Here, the authors present a 282 kyr precipitation record from the Papua New Guinea coast and show that obliquity forcing plays a more important role than previously recognized.

## Graphical abstract

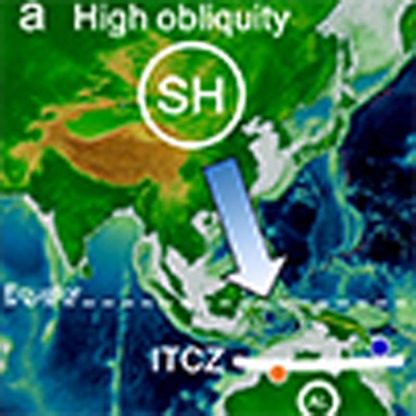

The Intertropical Convergence Zone (ITCZ) migrates meridionally with the seasonal angle of the sun^1^ and circles the globe in the tropics, marking the Earth's meteorological equator (Fig. 1). The convergence of inter-hemispheric trade winds leads to strong convective clouds, heavy precipitation and intense latent-heat transfer, dominating rainfall patterns worldwide. Owing to its intensive rainfall gradient, a small displacement in the position of the ITCZ can cause dramatic changes in hydrology and the frequency of extreme weather events—such as droughts, floods and tropical cyclones^2^. The collapse of the Mayan civilization and several Chinese Dynasties has been attributed to persistent droughts associated with ITCZ migrations^3^,^4^. The current build-up of atmospheric greenhouse gases has the potential to affect the future position of the ITCZ and corresponding climate^5^. An in-depth reconstruction of the position, structure and migration of the ITCZ is thus critical to our understanding of global climate and sustainable human socioeconomic development.

Lines of evidence from the past 210–220 kyr in Asian and American monsoon records^6^,^7^ suggest that the ITCZ was predominately driven by precessional forcing (∼20 kyr). Within the ITCZ territory, short-term terrestrial^8^,^9^,^10^ and marine^11^,^12^ proxy records have been reported. Few 100s-kyr records^13^ from the meteorological core of the ITCZ in the low-latitude Pacific, the southern counterpart of East Asia, severely hinders our understanding of the natural ITCZ variability related to orbital forcings in the Quaternary. Knowledge of the past variability of western Pacific ITCZ has global significance because this region is the largest heat and moisture source in the world.

Papua New Guinea (PNG), a mountainous terrain located at the southern border of the ITCZ (Fig. 1 and Supplementary Fig. 1), delivers a large amount of suspended sediments and solutes to the adjacent oceans as a result of the prodigious precipitation (>2,000 mm yr^−1^) in the region^14^,^15^. This transport occurs mostly in the wet season (>90% annual load) when the ITCZ is located over PNG^16^. Sediment archives from nearby marine basins, therefore, reflect this fluvial delivery and provide important information on precipitation related to the ITCZ position.

Relatively high rare earth element (REE) contents (for example, Nd ∼30 μg g^−1^) are characteristic of solid crustal materials, as compared with dissolved riverine (for example, ∼30 ng g^−1^) or seawater values (<1 pg g^−1^)^17^. This feature has led to the study of these elements and isotopes as terrestrial sediment tracers^17^,^18^,^19^ in the ocean.

Using inductively coupled plasma sector field mass spectrometric (ICP-SF-MS) techniques with 2*σ* precision of ±2–6% (ref. 20), we establish a 282-kyr-long record of REEs to calcium (REE/Ca) ratios in the planktonic foraminifera *Globigerinoides ruber* (Supplementary Fig. 2). The foraminiferal tests were sampled from a marine sediment core MD05-2925 (9°21′S, 151°28′E; water depth 1,661 m; Fig. 1 and Supplementary Fig. 1), collected 50 km off southeastern PNG to reveal the orbital-scale evolution of ITCZ precipitation intensity. Further, new calculations from a previous orbital-accelerated transient experiment^21^,^22^ using a coupled fast ocean-atmosphere model (FOAM) forced by variations in orbital parameters (see Methods for details) are conducted to offer clues of possible dynamical ITCZ migration processes in the western Pacific. Our geochemical records and modelling results reveal an important influence of obliquity forcing on the western Pacific ITCZ variability.

## Results

### Planktonic foraminiferal REE

Marine carbonates can record seawater REE composition even though seawater REE contents vary by an order of magnitude^23^,^24^. Planktonic foraminifera^23^ from the eastern Pacific and coral carbonates^24^ from the High Island of the Great Barrier Reef (GBR) have typical seawater REE patterns^25^, characterized by shale-normalized (SN)^26^ light (LREE, La-Nd) and middle REE (MREE, Sm-Ho) depletions, and heavy REE (HREE, Er-Lu) enrichments (Nd/Yb_SN_=0.17–0.28 and Gd/Yb_SN_=0.41–0.70; Fig. 2c,f,g). Deviating from seawater^25^ and marine carbonates^23^,^24^ from the open ocean, the REE patterns for the foraminiferal carbonates at the study site are characterized by enrichments of LREE and MREE (average Nd/Yb_SN_=0.45±0.04 (1 s.d. of the mean, *σ*_m_) and Gd/Yb_SN_=0.88±0.06 (1*σ*_m_)) and high REE concentrations (Fig. 2a), resembling more like the composition of PNG coastal seawater^27^ (Nd/Yb_SN_=0.31 and Gd/Yb_SN_=0.83; Fig. 2c). The local MREE-enriched source^17^ (Fig. 2b) combined with the REE fractionations in seawater^28^ produces the pattern illustrated in Fig. 2a. These features are consistent with dominance of river input to the upper water column as recorded in PNG coastal corals from Misima Island^29^ (Fig. 2e), which is termed PNG ‘island-weathering signature' supplied by precipitation-dependent river runoff^17^. The observations of an oxidative state at this core site, the absence of the formation of Mn-Fe oxides (see Methods) and no correlation between foraminiferal Nd/Ca and Fe/Ca data (Supplementary Figs 3 and 4), also support the assertion that the cleaned planktonic foraminifer REE can reliably capture the sea surface water condition.

There is no significant difference between REE patterns for periods with low and high foraminiferal REE contents over the entire MD05-2925 record (Supplementary Fig. 5). Only 10–20% variation of Nd/Yb_SN_ and Gd/Yb_SN_ ratios shows stable LREE/HREE and MREE/HREE ratios over the entire sequence (Supplementary Figs 4 and 5). This temporally consistent REE pattern shows that a terrestrial source is dominant in the record (see Supplementary Note 1 for detailed evaluation of the controls on foraminiferal REE/Ca). The implication is also supported by Nd isotopic data (Supplementary Fig. 2 and Supplementary Table 2) and a replicated record from an adjacent marine sedimentary core, ODP-1115B (9°11′S, 151°34′E; water depth 1,149 m) (Supplementary Note 1 and Supplementary Fig. 6). Therefore, the down-core planktonic foraminiferal REE/Ca sequence at MD05-2925 site can reflect the river runoff flux and be used as a qualitative proxy record of past ITCZ-related precipitation over PNG. All measured *G. ruber* REEs/Ca ratios (Supplementary Data 1) of core MD05-2925 consistently co-vary with a high correlation coefficient of >0.97 (except for Ce) over the past 282 kyr (Supplementary Fig. 2). Here we use Nd/Ca time series (Fig. 3c) to represent REE variability and to infer regional ITCZ-related precipitation changes.

### Inferred precipitation records

The MD05-2925 Nd/Ca sequence can be characterized by a sinusoidal-like curve with low values of 0.2–0.3 μmol mol^−1^ and 10 s-kyr wide peaks of 0.4–1.2 μmol mol^−1^. *G. ruber* Nd/Ca cycles are generally aligned with changes in precession-dominated Southern Hemisphere (SH) summer insolation values (Fig. 3c,e and Supplementary Fig. 7a). The agreement indicates that PNG precipitation variations are broadly driven by precessional forcing. Intense PNG precipitation results from the large temperature gradient between land and ocean in response to high SH summer insolation.

Comparison of our Nd/Ca record with contemporaneous stacked Chinese stalagmite δ^18^O records^6^,^30^,^31^,^32^ over the past 282 kyr is illustrated in Fig. 3. The stalagmite δ^18^O record has been interpreted as a record of summer monsoon precipitation and Asian summer monsoon (ASM) intensity; with more negative (positive) stalagmite δ^18^O values indicating higher (lower) precipitation/stronger (weaker) ASM intensities^30^,^31^. High foraminiferal Nd/Ca-inferred wet periods at PNG generally match positive stalagmite δ^18^O-derived dry conditions in mainland China and vice versa (Fig. 3b,c). This interhemispheric precipitation anti-phasing over the Asia–Pacific realm can be attributed to latitudinal shifts of the ITCZ and associated rain belts, driven by precession-dominated changes in seasonal insolation (Supplementary Fig. 7a).

In China, cave record-inferred precession-dominated precipitation intensity co-varies with solar radiation^6^ (Fig. 3a,b). However, our planktonic foraminiferal REE/Ca series (Fig. 3c) shows that precession is not the only orbital forcing mechanism operating on the ITCZ in the southern low-latitude Pacific. In PNG, there are six incompatible periods of low Nd/Ca-inferred precipitation at about 45, 90, 140, 165, 210 and 250 kyr BP (highlighted with grey bars in Fig. 3), when the Earth's axial tilt was high (Fig. 3c,f). Modelling results, synchronous with our proxy sequence (Fig. 3d and Supplementary Figs 8 and 9), also show consistent suppressed summer precipitation over PNG in the SH tropics at high obliquity periods. Indeed, spectral power analysis indicates that our foraminiferal Nd/Ca time series is dominated by obliquity periodicity (Supplementary Fig. 7d), highlighting the important role of Earth's axial tilt in modulating precipitation in the region of PNG.

## Discussion

The obliquity effect on SH tropical Pacific precipitation is most likely associated with its control on the meridional thermal-pressure contrast. Modelling results by FOAM suggest that high obliquity is responsible for the establishment of a strong Siberian high cell (Supplementary Fig. 8a) and East Asian winter monsoon system^22^,^33^,^34^. In an experiment using the Geophysical Fluid Dynamics Laboratory modelling, climate feedbacks and seasonal response may outcompete the local radiative forcing of obliquity and induce complicated response of northern high-latitude climate^35^. However, FOAM-inferred atmospheric response (Supplementary Fig. 8) is supported by other simulation results using Community Climate System Model version 3 (ref. 36; Supplementary Fig. 10) and Community Earth System version 1 (Supplementary Fig. 11).

**Figure 1:**
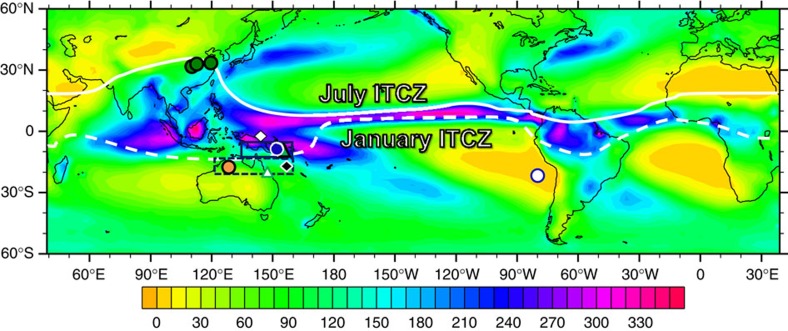
Precipitation map and study site. Map of mean annual precipitation (cm per year; 1988–2004; data source: http://jisao.washington.edu/data/gpcp/). Solid and dashed white lines represent the mean positions of the ITCZ in July and January, respectively. Symbols denote locations of Chinese caves^6^,^30^,^31^,^32^ (green circles), marine sediment cores MD05-2925 in this study (blue circle) and 54MC of ref. 23 (white circle), GBR coral^24^ (white triangle), PNG coastal coral^29^ (green triangle), PNG coastal seawater^27^ (white diamond), surface seawater of the Coral Sea^25^ (black diamond) and Gregory Lakes^38^ (orange circle). Simulated precipitation results in sectors of PNG (5–12°S and 130–160°E, blue solid lines) and North Australia (12–20°S and 120–160°E, blue dashed lines) are given in Fig. 3 and Supplementary Fig. 8.

Similar with the Siberian high, the Australian low, the counterpart of the meridional circulation loop, is also affected by obliquity (Supplementary Fig. 8c). Although, precessional forcing dominates local land-ocean thermal contrasts and influences the Australian low, the simulated Australian low does not rigidly follow precession. Extreme low pressures are always induced by high obliquity during the past 282 kyr and distinguishably stronger than those induced by precession (Supplementary Fig. 8). Obliquity-induced meridional circulation can affect the intensity of the Australian summer monsoon, the hemispheric counterpart of the Asian winter monsoon, through a cross-equatorial ‘pressure-push' process^37^. Specifically at high obliquity, a strong pressure gradient between an intensified Siberian high and Australian low enhances cross-equatorial flow of northerly winds (Supplementary Fig. 8a–c). Similar with the Australian low, the relative intensity of peak northerly winds at high obliquity are significantly raised, although the precession cycle is still obvious in the wind change, attributed to the effect of local thermal contrast. The enlarged peak northerly winds subsequently reinforce the southward shift of the ITCZ rain belt to its southernmost position (Fig. 4a). This northward/southward shift of the ITCZ leaves distinct rainfall patterns in different locations. The net effect is to increase precipitation in North Australia (Supplementary Fig. 8f) with compensated amounts in PNG at the six periods tagged in Fig. 3. The precipitation, thus, is relatively reduced at PNG despite high seasonal insolation and presents a stronger obliquity component (Supplementary Fig. 7). The intensified obliquity cycle in modelling PNG precipitation is qualitatively in agreement with our reconstruction (Fig. 3c,d). Support for such a strong southward migration of the ITCZ by high obliquity also comes from a 100-kyr record at Gregory Lakes (20°15′S, 127°30′E), on the fringe of the desert in semi-arid northwestern Australia^38^ (Figs 1 and 4). The occurrence of two past high lake stands at 37–50 and 95–105 kyr BP (Fig. 4 of ref. 38) matches the high-obliquity window and provides a SH terrestrial complement to our marine record.

At low obliquity, the ‘pressure-push' forcing^37^, strengthened by the capacious Asian landmass, is weak and the northerly wind intensity and ITCZ shift tends to follow precession-dominated insolation. The peak northerly wind and Australian low occur at high precession (∼20, 70, 115, 185 and 230 kyr BP; Supplementary Fig. 8). However, these precession-induced changes are not more vigorous than ones at intervals with high obliquity (Supplementary Fig. 8). For the scenario with low obliquity and high precession, the northerly wind is not as strong as relative to high-obliquity and high-precession cases and the centre of the strong convergence rain belt stays relatively in the north. As a result, PNG experiences enormous rainfall during those times in response to precession-dominated local insolation.

In contrast, owing to the limited area of the Australian continent, the low pressure near the warm Tibetan Plateau (topographic forcing) in boreal summer is predominately driven by local insolation changes^34^. This results in a precession-controlled ITCZ shift in East Asia, as inferred from Chinese cave records (Fig. 3). The northern and southern branches of the ITCZ in the Asian-Pacific realm appear to respond differently to orbital insolation. This interhemispheric asymmetry of ITCZ movements is attributed to distinct land-sea configurations and topography.

Our planktonic foraminiferal REE record near PNG and FOAM-simulated data reveal that obliquity can shift the position of the ITCZ and operate in tandem with precessional forcing^6^,^7^. Given that the obliquity signal is stronger relative to precession in the Nd/Ca-inferred precipitation record than in the model simulation (Supplementary Fig. 7), our proposed obliquity-induced ‘pressure-push' mechanism might be more significant for both PNG and North Australia, which can further be clarified by additional new low-latitude proxy records and advanced model simulations. Understanding the dynamics of ITCZ migration in the low-latitude Pacific through the Quaternary glacial-interglacial oscillations is essential for deciphering the dynamics of past global climate. The prevalence of the obliquity signal in both ice volume^39^ and the low-latitude western Pacific as implicated in our precipitation record highlights that this orbital forcing plays an important role in global hydrologic cycles.

## Methods

### Core site

The selected marine sediment core, MD05-2925, is 2,843 cm in length and was recovered in June 2005 during the IMAGES XIII-PECTEN (Past Equatorial Climate: Tracking El Niño) cruise on board the R.V. Marion Dufresne of the French Polar Institute (IPEV). The core site is located at the southern margin of the Western Pacific Warm Pool, 110 km to Fergusson Island, 50 km off southeastern tip of PNG (Fig. 1 and Supplementary Fig. 1).

The core sediment is composed of a mixture of biogenic carbonate and silty clay^40^. The chlorophyll level of 0.2 mg m^−3^ (ref. 41) for surrounding surface water in eastern PNG suggests low regional productivity. The dissolved-oxygen concentrations are high (>3 ml l^−1^) through the whole water column including bottom waters of eastern PNG^42^. The local benthic oxygen flux, reflecting organic matter remineralization, is only 0.1 mol m^−2^ per year (ref. 43). It is lower than the values of 0.8 mol m^−2^ per year for the reducing margins (notably in the eastern boundary upwelling systems and North Indian Ocean)^43^. These data indicate an oxidative condition at this study site. The upper 1,510 cm was used in this study.

### Age model

The age model was established based on accelerator mass spectrometry (AMS) radiocarbon (^14^C) dates (Supplementary Table 1) and oxygen isotope stratigraphy (Supplementary Fig. 12). A series of planktonic foraminiferal AMS ^14^C dates at 19 different depths, including 200 individuals of *Globigerinoides sacculifer* (>500 μm) each, from the upper 292 cm of the core were measured. Dates were calibrated to calendar ages (before 1950 AD) using CALIB 6.0.1 software^44^ with a reservoir age difference (Δ*R*) estimated from the Marine Reservoir Correction Database (http://calib.qub.ac.uk/marine/). The calculated weighted mean Δ*R* value is 64±23 years for the selected four sites around the Solomon Sea^45^. The chronology was based on linear interpolation between calibrated ^14^C dates (Supplementary Table 1).

For the depths >292 cm, the age model was developed by correlating the composite benthic foraminiferal oxygen isotopic data of core MD05-2925 to the LR04 stack record^46^ (Supplementary Fig. 12). Composite benthic foraminiferal oxygen isotope data are established with benthic foraminifera (>250 μm, 2–4 individuals each depth), including the *Uvigerina spp.* (201 samples), *Cibicidoides wuellerstorfi* (11 samples) and *Bulimina spp.* (7 samples) at core depths of 157–1,897 cm (Supplementary Fig. 13). Measurement of δ^18^O data, relative to Vienna Pee Dee Belemnite carbonate standard, was performed on a Micromass IsoPrime isotope ratio mass spectrometer with 1*σ* reproducibility of ±0.05‰ (ref. 47). δ^18^O offsets of *C. wuellerstorfi* (+0.64‰)^48^ and *Bulimina spp.* (−0.11‰)^49^ from *Uvigerina spp.* were corrected. This age model is supported by the last occurrence of *G. ruber* (pink) occurred at depths of 830–835 cm, corresponding to 129.8 kyr BP (Supplementary Fig. 12), consistent with the observation in the southern South China Sea^50^.

### Screening for diagenesis

Scanning electron microscopy images of 30 uncleaned individuals of planktonic foraminifera *G. ruber* (white, *s.s.* 250–300 μm) at six depths of 477 (50.1 kyr BP) and 617 cm (81.6 kyr BP) with low REE content, 527 (56.8 kyr BP) and 577 cm (73.2 kry BP) with high REE content and 877 (135.0 kyr BP) and 917 cm (146.1 kyr BP) with moderate REE content (Supplementary Fig. 14) were carefully screened. Thirty more uncleaned individuals picked from six depths (87, 267, 787, 1,087, 1,317 and 1,477 cm), respectively, at marine isotope stages 1, 2, 5, 6, 7 and 8 were also checked with scanning electron microscopy. No nodules of Mn-Fe oxides were noticeable and all shell walls were intact and primitive (Supplementary Fig. 14). Additional careful inspection under microscope did not observe Mn-Fe oxides for 1,200 tests from the selected 12 depths. For conservative consideration, we still applied a full cleaning procedure on all samples.

### Measurement of foraminiferal trace elements

REE contents of down-core planktonic foraminifera *G. ruber* (white, *s.s.* 250–300 μm) were measured (Supplementary Fig. 2). Although no Mn-Fe nodules were noticeable (Supplementary Fig. 14), *G. ruber* tests were cleaned with a full cleaning procedure for foraminiferal trace metal analysis, modified from refs 51, 52. About 20 foraminiferal individuals were gently crushed, placed in a Teflon vial and washed sequentially with the following reagents (all at pH 8.5–9.0): (i) ethanol+H_2_O, (ii) 1% H_2_O_2_, (iii) 0.56 M NH_4_Cl and (iv) 0.43 M NH_2_OH. Cleaned tests, polished with 10^−3^ M HNO_3_ to dissolve a possible thin post-depositional magnesium-rich surface layer^51^,^52^, were rinsed with ultrapure water three times to wash off the residues of chemicals and then dissolved in 5% HNO_3_ for instrumental analyses. All chemical procedures were performed on a class-100 laminar-flow bench in a class-10,000 clean room in the High-precision Mass Spectrometry and Environment Change Laboratory (HISPEC), Department of Geosciences, National Taiwan University.

REE/Ca ratios were calculated using the ion beams of ^46^Ca, ^139^La, ^140^Ce, ^141^Pr, ^146^Nd, ^147^Sm, ^153^Eu, ^160^Gd, ^159^Tb, ^163^Dy, ^165^Ho, ^166^Er, ^172^Yb and ^175^Lu, detected on an ICP-SF-MS, Thermo Fisher ELEMENT II, equipped with a dry introduction Cetac ARIDUS^20^ system. Two-month 2*σ* reproducibility is ±1.9–6.5%. Mg/Ca, Mn/Ca and Fe/Ca ratios with respective 2*σ* errors of ±0.23%, ±0.68% and ±2.7% were determined on the same ICP-SF-MS, equipped with a quartz Scott-type double-pass spray chamber^53^.

**Figure 2:**
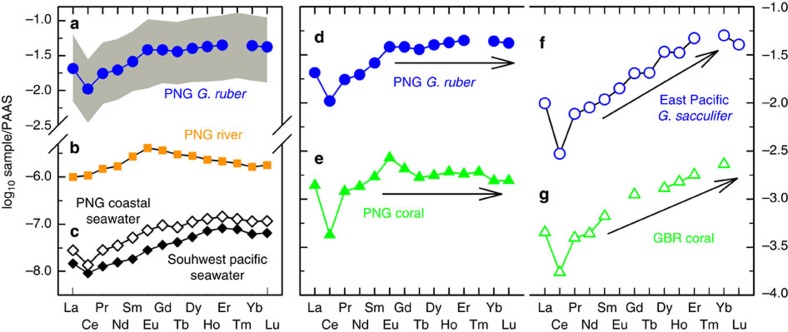
REE patterns of waters and marine carbonates. (**a**) Averaged MD05-2925 *G. ruber* data with temporal variability range over the past 282 kyr in this study (grey area). (**b**) PNG river^17^. (**c**) PNG coastal seawater (depth of 40 m at station EUC-Fe 27 from ref. 27, hollow diamonds) and open-ocean surface seawater of the Coral Sea in the southwest Pacific (depth of 0–200 m at station SA-7 from ref. 25, black diamonds). Comparison of REE patterns from (**d**) MD05-2925 *G. ruber* in this study (blue circles), (**e**) PNG coastal coral^29^ (green triangles), (**f**) East Pacific core-top planktonic foraminifera *G. sacculifer* (site 54MC of ref. 23; hollow circles), and (**g**) GBR coral^24^ (hollow triangles). Site locations are plotted in Fig. 1 and Supplementary Fig. 1. Arrows depict the trend of the REE patterns. The REEs are shale normalized^26^.

**Figure 3:**
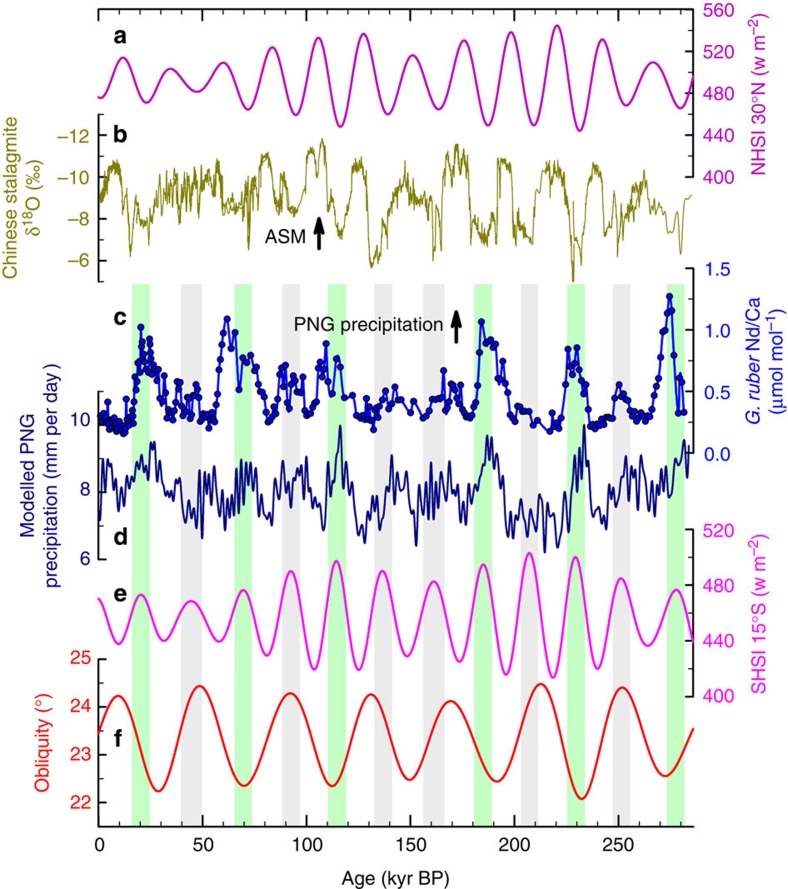
Comparison of PNG planktonic foraminifera *G. ruber* Nd/Ca from MD05-2925 with other records over the past 282 kyr. (**a**) Northern hemisphere summer insolation (NHSI, 15 July) at 30°N (ref. 61). (**b**) Stacked δ^18^O of Chinese stalagmites^6^,^30^,^31^,^32^. (**c**) MD05-2925 *G. ruber* Nd/Ca (2*σ* precision: ±2.6%, ref. 20). (**d**) Modelled PNG precipitation (5–12°S and 130–160°E). (**e**) Southern hemisphere summer insolation (SHSI, 15 January) at 15°S (ref. 61). (**f**) Earth obliquity^61^. Arrows depict an increase of the ASM and the foraminifera-inferred PNG precipitation. PNG precipitation is intensified at six periods with high SHSI (vertical grass green bars with Nd/Ca >0.75 μmol mol^−1^), but does not closely reflect high SHSI at six other intervals (grey bars).

**Figure 4:**
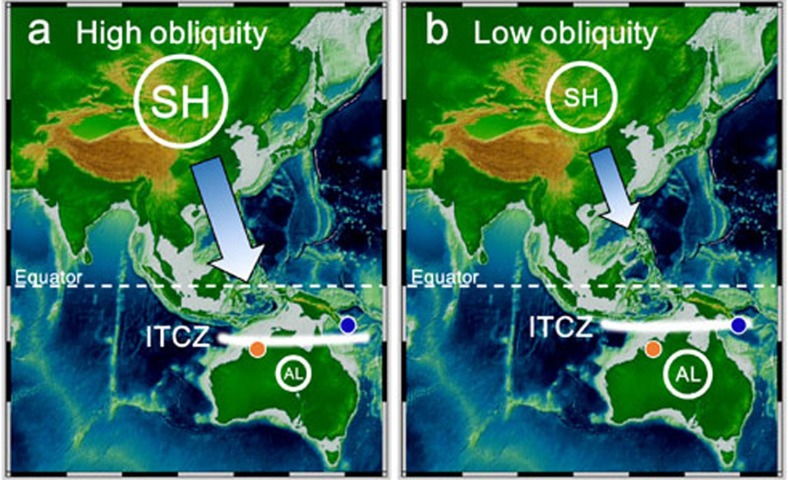
Illustration of the proposed pressure-push mechanism driving the southern branch of the western Pacific ITCZ in the Asia-Pacific realm. This illustration is based on the condition of high Southern Hemisphere summer insolation (high precession). (**a**) High obliquity. A strong pressure gradient between intensified SH and AL enhances cross-equatorial and flow reinforces the southward shift of the ITCZ rain belt to the southernmost position in boreal winter. The net effect is to increase precipitation in North Australia, with compensated amounts in PNG. (**b**) Low obliquity. The cross-equatorial ‘pressure-push' forcing is weak at low-obliquity and the centre of ITCZ rain belt stays in the north, relative to high-obliquity cases in boreal winter. As a result, PNG experiences enormous rainfall while North Australia receives less rainfall.AL, Australian low; SH, Siberian high; light blue arrow, surface wind intensity; solid white line, core position of the western Pacific ITCZ^1^; blue circle, marine sediment core MD05-2925 site; brown circle, Gregory Lakes^38^.

An insignificant correlation between Mg/Ca and Fe/Ca data (Supplementary Fig. 3a) indicates the effectiveness of the cleaning techniques. Moreover, the measured REE/Ca patterns (Fig. 2) are different from shale-like patterns for uncleaned foraminifera with greater light REE (LREE) contents enrichment and unclear Ce anomalies^54^. We also tested our cleaning procedure/analytical technique by an interlaboratory comparison for analysing REE/Ca ratios of benthic foraminifera *C. wuellerstorfi* sample from core GGC-15 (ref. 20). The results showed that our REE data replicate measurements using a REE cleaning method at the University of Cambridge (Fig. 5 of ref. 20). Detailed instrumentation and fidelity of our methodology for foraminiferal test REE/Ca determination are described in ref. 20.

### Nd isotopic measurement

Planktonic foraminifera *G. ruber* and sediment (<63 μm) samples were collected from two depth intervals of 472–477 cm (49.5–50.1 kyr BP, 580 individuals, >250 μm) and 537–542 cm (58.8–60.6 kyr BP, 250 individuals, >250 μm) of core MD05-2925 (Supplementary Fig. 2). The picked planktonic foraminifera samples were cleaned with the same protocol for REE/Ca ratio analysis and then dissolved in 2 M HNO_3_. The sediment samples were first cleaned with 10% CH_3_COOH to remove carbonate, and subsequently cleaned with a reductive reagent (1 M NH_2_OH·HCl in 25% CH_3_COOH) to remove possible Fe-Mn phases on the sample surface^55^. The cleaned sediment samples were decomposed in a mixed solution of HF, HClO_4_ and HNO_3_, and then dissolved in 2 M HNO_3_.

Neodymium in the 2 M HNO_3_ dissolved samples was extracted by a two-stage column separation^56^. The REE fraction in the solution was purified from the remaining major and trace elements using Eichrom RE resin. Neodymium was subsequently separated from the other REE with Eichrom Ln resin.

Neodymium isotopic compositions were measured by a multi-collector ICP-MS, Thermo Fisher Neptune, in the HISPEC. The measured ^143^Nd/^144^Nd ratios were normalized to ^146^Nd/^144^Nd=0.7219 using an exponential law. La Jolla standard was measured at 0.511811±0.000014 (or ±0.27 *ɛ*; 2*σ*, *n*=13). All ^143^Nd/^144^Nd ratios were calibrated to the reported value relative to the La Jolla standard value of 0.511858 (ref. 57). Sample ^143^Nd/^144^Nd ratios [(^143^Nd/^144^Nd)_sample_] are expressed as *ɛ* notation defined by an equation of *ɛ*_Nd_=[(^143^Nd/^144^Nd)_sample/_(^143^Nd/^144^Nd)_CHUR_−1] × 10^4^, where the ^143^Nd/^144^Nd ratio of CHUR standard for Chondritic Uniform Reservoir [(^143^Nd/^144^Nd)_CHUR_] is 0.512638 (ref. 58).

### Modelling simulation

The simulated precipitation and climatological records used in this study are from an orbital-accelerated transient run using FOAM conducted by Kutzbach *et al.*^21^ and re-analysed by Shi *et al.*^22^. FOAM, a fully coupled, mixed-resolution, and high-throughput general circulation model, provides a good simulation of mean condition and variability^59^. With a factor of 100, FOAM was integrated for 2,820 years under orbital forcing only to obtain climate evolution over the past 282 kyr. Changes in global ice volume/sea level and greenhouse gases were not considered. The spatial resolution is set to 4° × 7.5° for atmosphere and 1.4° × 2.8° for ocean. Because of the limitation of orbital acceleration, it is difficult for the deep ocean to reach equilibrium so that the full potential of the deep ocean feedback cannot be achieved. However, in previous studies^21^,^22^,^60^, the responses of monsoon precipitation, mostly considered as a response to the changes in the atmosphere-surface ocean system, to the orbital insolation can be successfully retrieved in the annual variability. A detailed description on the transient experiment is available in ref. 21.

## Additional information

**How to cite this article:** Liu, Y. *et al.* Obliquity pacing of the western Pacific Intertropical Convergence Zone over the past 282,000 years. *Nat. Commun.* 6:10018 doi: 10.1038/ncomms10018 (2015).

## Supplementary Material

Supplementary Information Supplementary Figures 1-14, Supplementary Tables 1-2, Supplementary Note 1 and Supplementary References.

Supplementary Data MD05-2925 foraminiferal trace metals and δ^18^O data over the past 282,000 years and ODP-1115B foraminiferal geochemical data

## References

[b1] Waliser D. E. & Gautier C. Satellite-derived climatology of the ITCZ. J. Clim 6, 2162–2174 (1993).

[b2] Cai W. *et al.* More extreme swings of the South Pacific convergence zone due to greenhouse warming. Nature 488, 365–369 (2012). 22895343 10.1038/nature11358

[b3] Haug G. H. *et al.* Climate and the collapse of Maya Civilization. Science 299, 1731–1735 (2003). 12637744 10.1126/science.1080444

[b4] Yancheva G. *et al.* Influence of the intertropical convergence zone on the East Asian monsoon. Nature 445, 74–77 (2007). 17203059 10.1038/nature05431

[b5] Sachs J. P. *et al.* Southward movement of the Pacific intertropical convergence zone AD 1400–1850. Nat. Geosci 2, 519–525 (2009).

[b6] Wang Y. J. *et al.* Millennial- and orbital-scale changes in the East Asian monsoon over the past 224,000 years. Nature 451, 1090–1093 (2008). 18305541 10.1038/nature06692

[b7] Wang X. F. *et al.* Wet periods in northeastern Brazil over the past 210 kyr linked to distant climate anomalies. Nature 432, 740–743 (2004). 15592409 10.1038/nature03067

[b8] Partin J. W., Cobb K. M., Adkins J. F., Clark B. & Fernandez D. P. Millennial-scale trends in west Pacific warm pool hydrology since the Last Glacial Maximum. Nature 449, 452–456 (2007). 17898765 10.1038/nature06164

[b9] Griffiths M. L. *et al.* Increasing Australian–Indonesian monsoon rainfall linked to early Holocene sea-level rise. Nat. Geosci 2, 636–639 (2009).

[b10] Ayliffe L. K. *et al.* Rapid interhemispheric climate links via the Australasian monsoon during the last deglaciation. Nat. Commun 4, 2908 (2013). 24309539 10.1038/ncomms3908

[b11] Haug G. H., Hughen K. A., Sigman D. M., Peterson L. C. & Röhl U. Southward migration of the intertropical convergence zone through the Holocene. Science 293, 1304–1308 (2001). 11509727 10.1126/science.1059725

[b12] Mohtadi M. *et al.* Glacial to Holocene swings of the Australian–Indonesian monsoon. Nat. Geosci 4, 540–544 (2011).

[b13] Tachikawa K. *et al.* The precession phase of hydrological variability in the Western Pacific Warm Pool during the past 400 ka. Quat. Sci. Rev. 30, 3716–3727 (2011).

[b14] Milliman J. D., Farnsworth K. L. & Albertin C. S. Flux and fate of fluvial sediments leaving large islands in the East Indies. J. Sea Res. 41, 97–107 (1999).

[b15] Nittrouer C. A., Brunskill G. J. & Figueiredo A. G. Importance of tropical coastal environments. Geo-Mar. Lett. 15, 121–126 (1995).

[b16] Chappell N. A., Tych W., Shearman P., Lokes B. & Chitoa J. in Sediment Problems and Sediment Management in Asian River Basins ed. Walling D. E. 92–102IAHS Press (2011).

[b17] Sholkovitz E. R., Elderfield H., Szymczak R. & Casey K. Island weathering: river sources of rare earth elements to the Western Pacific Ocean. Mar. Chem. 68, 39–57 (1999).

[b18] Burton K. W. & Vance D. Glacial-interglacial variations in the neodymium isotope composition of seawater in the Bay of Bengal recorded by planktonic foraminifera. Earth Planet. Sci. Lett. 176, 425–441 (2000).

[b19] Stoll H. M., Vance D. & Arevalos A. Records of the Nd isotope composition of seawater from the Bay of Bengal: Implications for the impact of Northern Hemisphere cooling on ITCZ movement. Earth Planet. Sci. Lett. 255, 213–228 (2007).

[b20] Shen C.-C. *et al.* Measurements of natural carbonate rare earth elements in femtogram quantities by inductive coupled plasma sector field mass spectrometry. Anal. Chem. 83, 6842–6848 (2011). 21774547 10.1021/ac201736w

[b21] Kutzbach J. E., Liu X., Liu Z. & Chen G. Simulation of the evolutionary response of global summer monsoons to orbital forcing over the past 280,000 years. Clim. Dynam. 30, 567–579 (2008).

[b22] Shi Z. *et al.* Distinct responses of East Asian summer and winter monsoons to astronomical forcing. Clim. Past 7, 1363–1370 (2011).

[b23] Haley B. A., Klinkhammer G. P. & Mix A. C. Revisiting the rare earth elements in foraminiferal tests. Earth Planet. Sci. Lett. 239, 79–97 (2005).

[b24] Wyndham T., McCulloch M., Fallon S. & Alibert C. High-resolution coral records of rare earth elements in coastal seawater: biogeochemical cycling and a new environmental proxy. Geochim. Cosmochim. Acta 68, 2067–2080 (2004).

[b25] Zhang J. & Nozaki Y. Rare earth elements and yttrium in seawater: ICP-MS determinations in the East Caroline, Coral Sea, and South Fiji basins of the western South Pacific Ocean. Geochim. Cosmochim. Acta 60, 4631–4644 (1996).

[b26] McLennan S. M. Rare earth elements in sedimentary rocks; influence of provenance and sedimentary processes. Rev. Mineral. Geochem. 21, 169–200 (1989).

[b27] Grenier M. *et al.* From the subtropics to the central equatorial Pacific Ocean: Neodymium isotopic composition and rare earth element concentration variations. J. Geophys. Res.-Oceans 118, 592–618 (2013).

[b28] Sholkovitz E. R., Landing W. M. & Lewis B. L. Ocean particle chemistry: the fractionation of rare earth elements between suspended particles and seawater. Geochim. Cosmochima. Acta 58, 1567–1579 (1994).

[b29] Fallon S. J., White J. C. & McCulloch M. *Porites* corals as recorders of mining and environmental impacts: Misima Island, Papua New Guinea. Geochim. Cosmochima. Acta 66, 45–62 (2002).

[b30] Wang Y. J. *et al.* A high-resolution absolute-dated late Pleistocene monsoon record from Hulu Cave, China. Science 294, 2345–2348 (2001). 11743199 10.1126/science.1064618

[b31] Cheng H. *et al.* A penultimate glacial monsoon record from Hulu Cave and two-phase glacial terminations. Geology 34, 217–220 (2006).

[b32] Cheng H. *et al.* Ice age terminations. Science 326, 248–252 (2009). 19815769 10.1126/science.1177840

[b33] Lee S. & Poulsen C. J. Tropical Pacific climate response to obliquity forcing in the Pleistocene. Paleoceanography 20, PA4010 (2005).

[b34] Wyrwoll K.-H., Liu Z., Chen G., Kutzbach J. E. & Liu X. Sensitivity of the Australian summer monsoon to tilt and precession forcing. Quat. Sci. Rev. 26, 3043–3057 (2007).

[b35] Erb M. P., Broccoli A. J. & Clement A. C. The contributionof radiative feedbacks to orbitally-driven climate change. J. Clim 26, 5897–5914 (2013).

[b36] Li X., Liu X., Qiu L., An Z. & Yin Z. Transient simulation of orbital-scale precipitation variation in monsoonal East Asia and arid central Asia during the last 150 ka. J. Geophys. Res. Atmos 118, 7481–7488 (2013).

[b37] An Z. S. The history and variability of the East Asian paleomonsoon climate. Quat. Sci. Rev. 19, 171–187 (2000).

[b38] Fitzsimmons K. E., Miller G. H., Spooner N. A. & Magee J. W. Aridity in the monsoon zone as indicated by desert dune formation in the Gregory Lakes basin, northwestern Australia. Aust. J. Earth Sci. 59, 469–478 (2012).

[b39] Raymo M. E. & Nisancioglu K. The 41 kyr world: Milankovitch's other unsolved mystery. Paleoceanography 18, 1011 doi:10.1029/2002PA000791 (2003).

[b40] Beaufort L., Droxler A., Chen M., Yokoyama Y., Balut Y. & Rothe S. MD148-PECTEN IMAGES XIII cruise report, *Inst. Pol. Fr., Plouzaně, France* (2005).

[b41] Radenac M., Léger F., Singh A. & Delcroix T. Sea surface chlorophyll signature in the tropical Pacific during eastern and central Pacific ENSO events. J. Geophys. Res. 117, C04007 (2012).

[b42] Garcia H. E. *et al.* in *World Ocean Atlas 2009*, (ed. Levitus, S.) NOAA Atlas NESDIS 70, 344 (U.S. Government Printing Office, 2010).

[b43] Jahnke R. A. Benthic oxygen fluxes. JGOFS Rep. 38, 17 (2003).

[b44] Stuiver M., Reimer P. J. & Reimer R. W. CALIB 6.0, WWW program and documentation (2010).

[b45] Petchey F., Phelan M. & White P. J. New ΔR values for the Southwest Pacific Ocean. Radiocarbon 46, 1005–1014 (2004).

[b46] Lisiecki L. E. & Raymo M. E. Pliocene-Pleistocene stack of 57 globally distributed benthic δ^18^O records. Paleoceanography 20, PA1003 (2005).

[b47] Lo L. *et al.* Persistent sea surface temperature and declined sea surface salinity in the northwestern tropical Pacific over the past 7500 years. J. Asian Earth Sci. 66, 234–239 (2013).

[b48] Shackleton N. J. & Opdyke N. D. Oxygen isotope and palaeomagnetic stratigraphy of Equatorial Pacific core V28-238: Oxygen isotope temperatures and ice volumes on a 10^5^ year and 10^6^ year scale. Quat. Res 3, 39–55 (1973).

[b49] Oba T. *et al.* Paleoceanographic change off central Japan since the last 144,000 years based on high-resolution oxygen and carbon isotope records. Global Planet. Change 53, 5–20 (2006).

[b50] Lee M. Y., Wei K. Y. & Chen Y. G. High resolution oxygen isotope stratigraphy for the last 150,000 years in the southern South China Sea: Core MD972151. Terr. Atmos. Ocean. Sci. 10, 239–254 (1999).

[b51] Shen C.-C. *et al.* High precision glacial-interglacial benthic foraminiferal Sr/Ca records from the eastern equatorial Atlantic Ocean and Caribbean Sea. Earth Planet. Sci. Lett. 190, 197–209 (2001).

[b52] Pena L. D., Calvo E., Cacho I., Eggins S. & Pelejero C. Identification and removal of Mn-Mg-rich contaminant phases on foraminiferal tests: Implications for Mg/Ca past temperature reconstructions. Geochem. Geophys. Geosys 6, QO9P02 (2005).

[b53] Lo L. *et al.* Determination of element/Ca ratios in foraminifera and corals using cold- and hot-plasma techniques in inductively coupled plasma sector field mass spectrometry. J. Asian Earth Sci. 81, 115–122 (2014).

[b54] Pena L. D. *et al.* Rapid changes in meridional advection of Southern Ocean intermediate waters to the tropical Pacific during the last 30 kyr. Earth Planet. Sci. Lett. 368, 20–32 (2013).

[b55] Bayon G. *et al.* An improved method for extracting marine sediment fractions and its application to Sr and Nd isotopic analysis. Chem. Geol. 187, 179–199 (2002).

[b56] Pin C. & Zalduegui J. F. S. Sequential separation of light rare-earth elements, thorium and uranium by miniaturized extraction chromatography: Application to isotopic analyses of silicate rocks. Anal. Chim. Acta 339, 79–89 (1997).

[b57] Lugmair G. W., Shimamura T., Lewis R. S. & Anders E. Samarium-146 in the early solar system: evidence from neodymium in the Allende meteorite. Science 222, 1015–1018 (1983). 17776245 10.1126/science.222.4627.1015

[b58] Jacobsen S. B. & Wasserburg G. J. Sm–Nd isotopic evolution of chondrites. Earth Planet. Sci. Lett. 50, 139–155 (1980).

[b59] Jacob R. L. *et al.* Computational Design and Performance of the Fast Ocean Atmosphere Model, Version One. Proc. International Conference on Computational Science eds Alexandrov V. N., Dongarra J. J., Tan C. J. K. 175–184Springer-Verlag (2001).

[b60] Shi Z., Liu X. & Cheng X. Anti-phased response of northern and southern East Asian summer precipitation to ENSO modulation of orbital forcing. Quat. Sci. Rev. 40, 30–38 (2012).

[b61] Berger A. L. Long-term variations of caloric insolation resulting from the Earth's orbital elements. Quat. Res 9, 139–167 (1978).

