# Supplementary material for: Obliquity pacing of the western Pacific Intertropical Convergence Zone over the past 282,000 years
Source: Nat Commun. 2015 Nov 25;6:10018. doi: 10.1038/ncomms10018 (PMC4674685; doi:10.1038/ncomms10018)
Supplement: Supplementary Information — Supplementary Figures 1-14, Supplementary Tables 1-2, Supplementary Note 1 and Supplementary References. [file ncomms10018-s1.pdf]

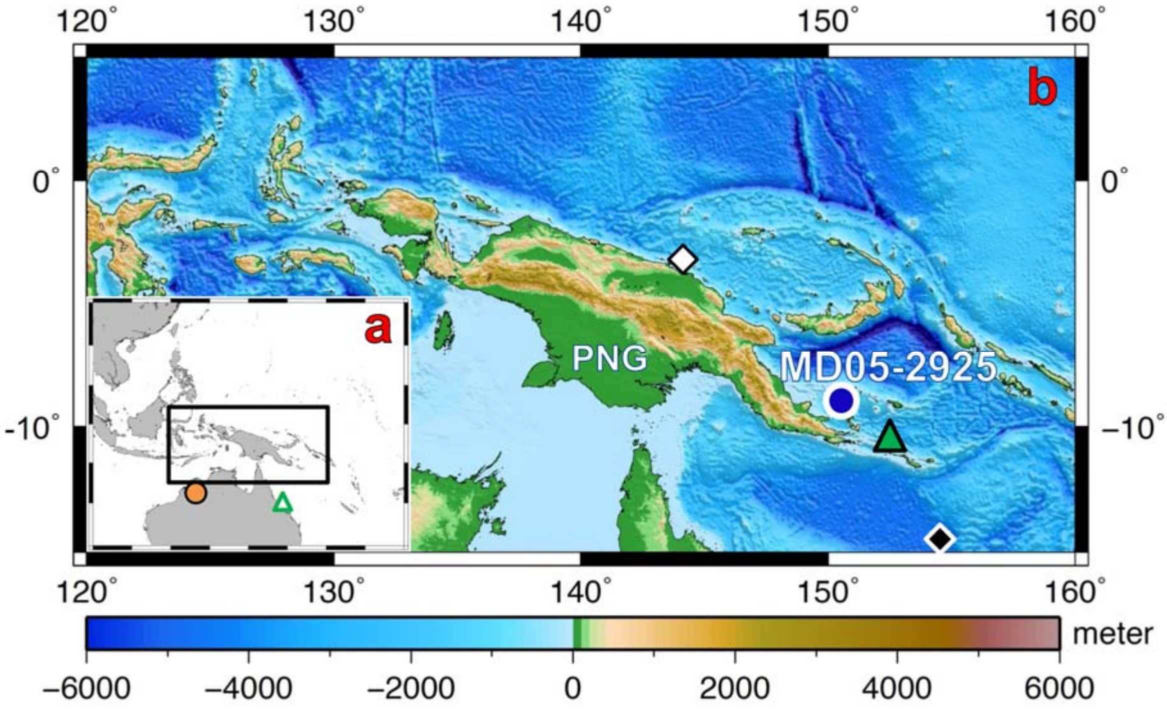

Supplementary Figure 1. Maps of (a) the western Pacific, and (b) topography of Papua New Guinea (PNG) and surrounding ocean basins. Symbols denote locations of the marine sediment core MD05-2925 in this study (blue circle), PNG coastal coral[16] (green triangle), PNG coastal seawater[7] (white diamond), surface seawater of the Coral Sea[17] (black diamond), Great Barrier Reef coral[18] (white triangle), and Gregory Lakes[19] (brown).

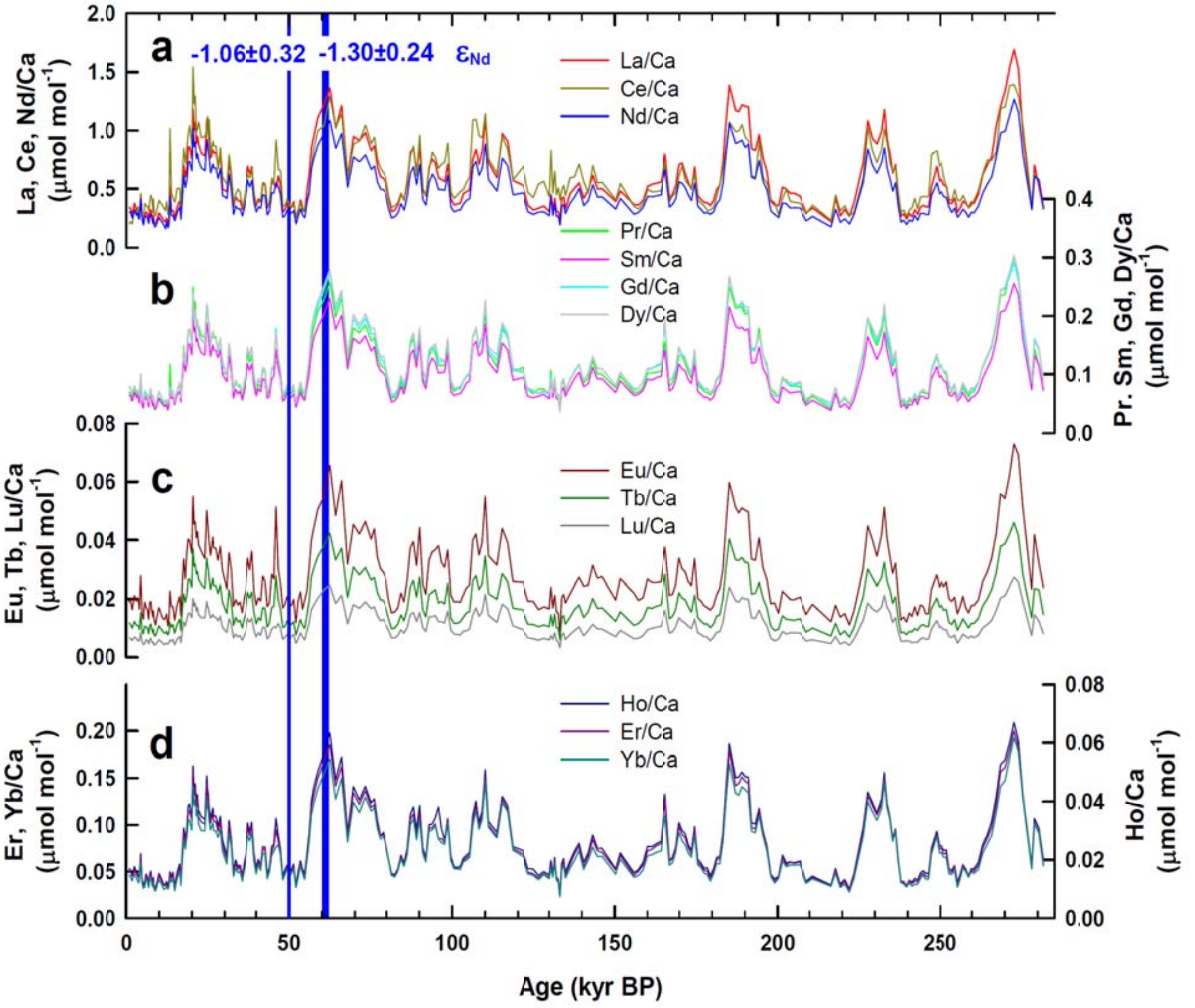

Supplementary Figure 2. Time series of REE/Ca of planktonic foraminifera *G. ruber*. **(a)** La/Ca, Ce/Ca and Nd/Ca. **(b)** Pr/Ca, Sm/Ca, Gd/Ca and Dy/Ca. **(c)** Eu/Ca ,Tb/Ca and Lu/Ca. **(d)** Ho/Ca, Er/Ca and Yb/Ca. ε_{Nd} values at ages of 49.5-50.1 and 58.8-60.6 kyr BP are given in panel **(a)**.

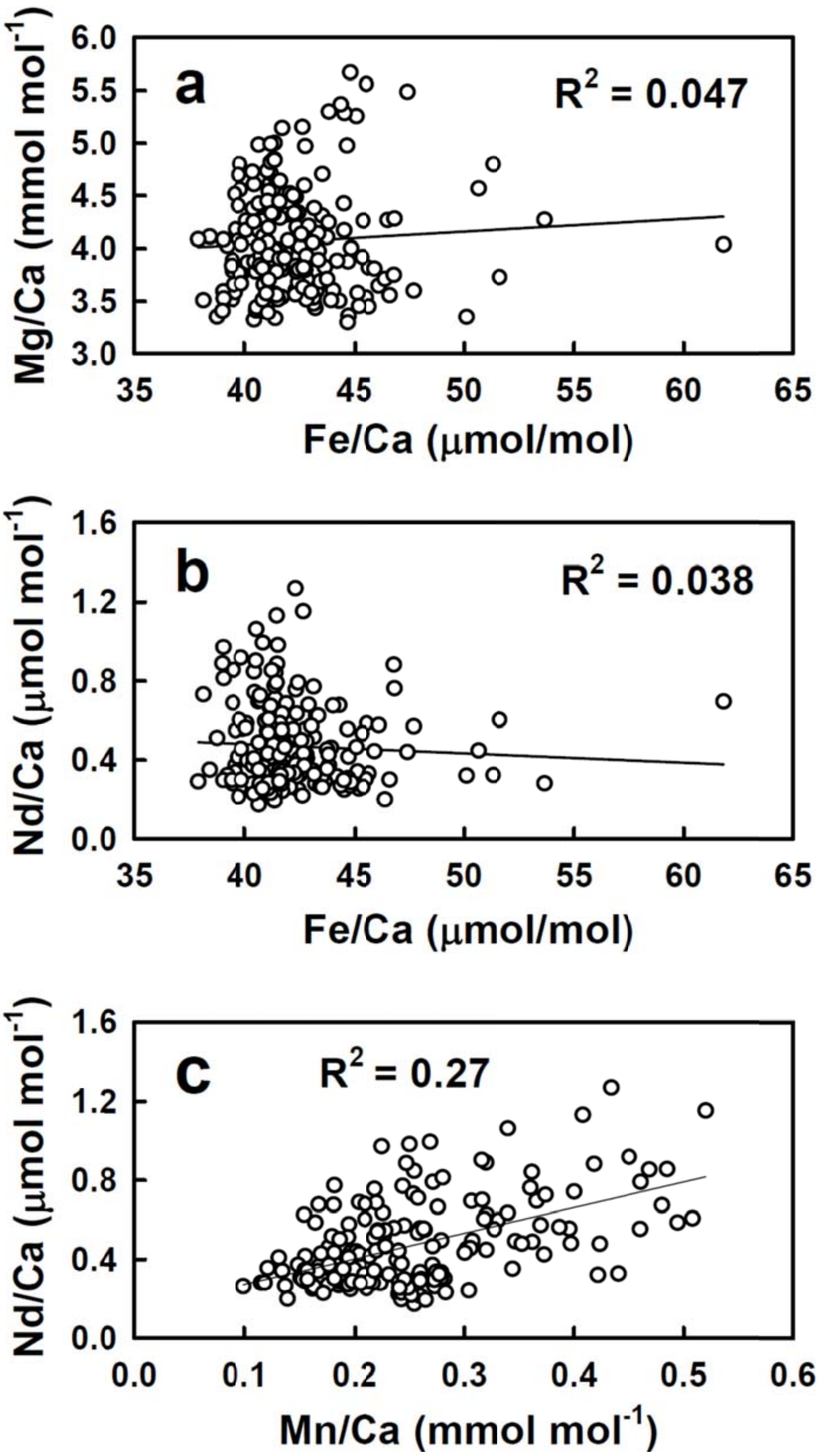

Supplementary Figure 3. Plots of MD05-2925 *G. ruber* (a) Mg/Ca vs. Fe/Ca, **(b)** Nd/Ca vs. Fe/Ca, and **(c)** Nd/Ca vs. Mn/Ca.

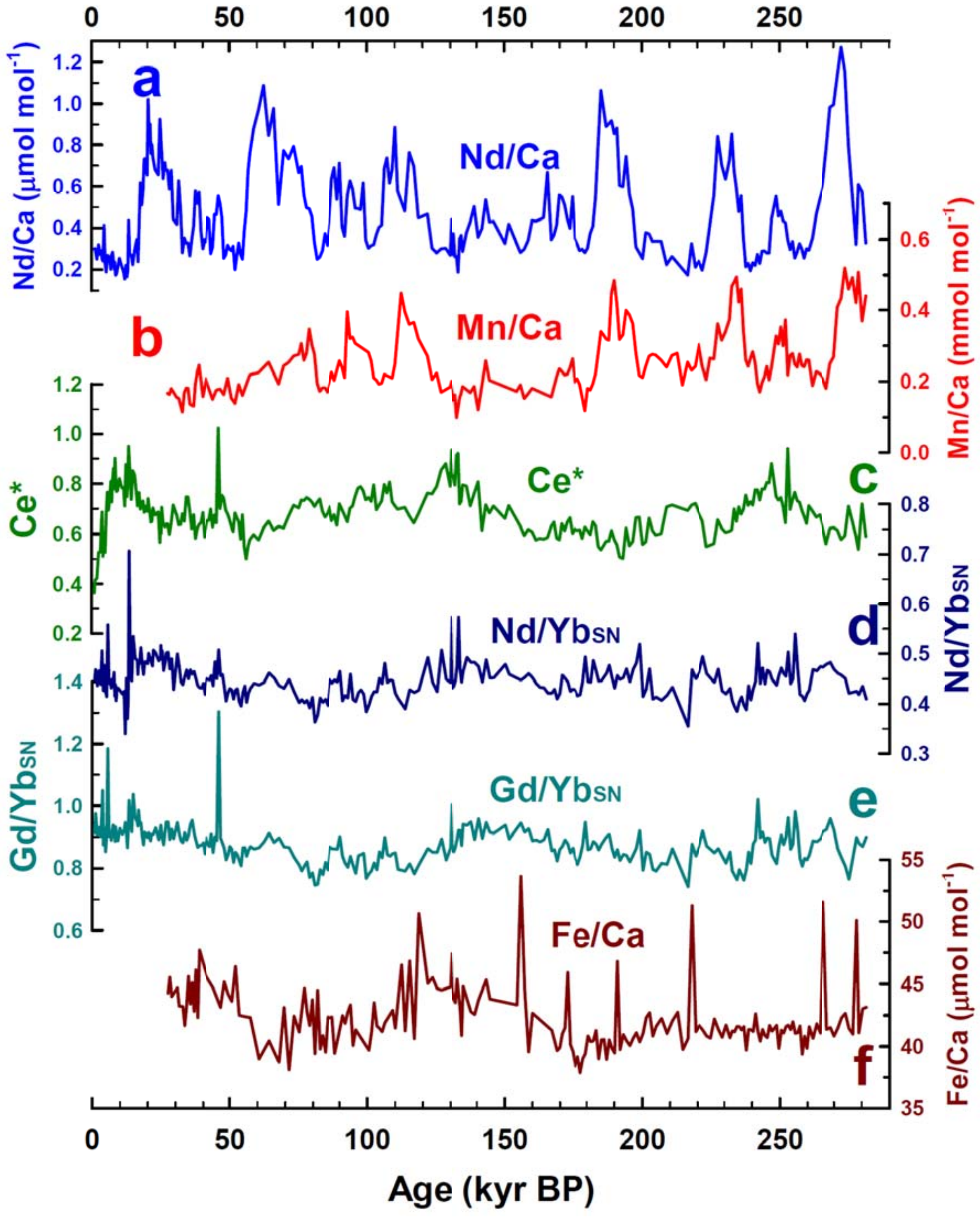

Supplementary Figure 4. Time series of trace elemental patterns of MD05-2925 *G. ruber*. (a) Nd/Ca. **(b)** Mn/Ca. **(c)** Ce* [Ce* = $(3Ce_{SN})/(2La_{SN}+Nd_{SN})$]. **(d)** Nd/Yb_{SN}. **(e)** Gd/Yb_{SN}. **(f)** Fe/Ca.

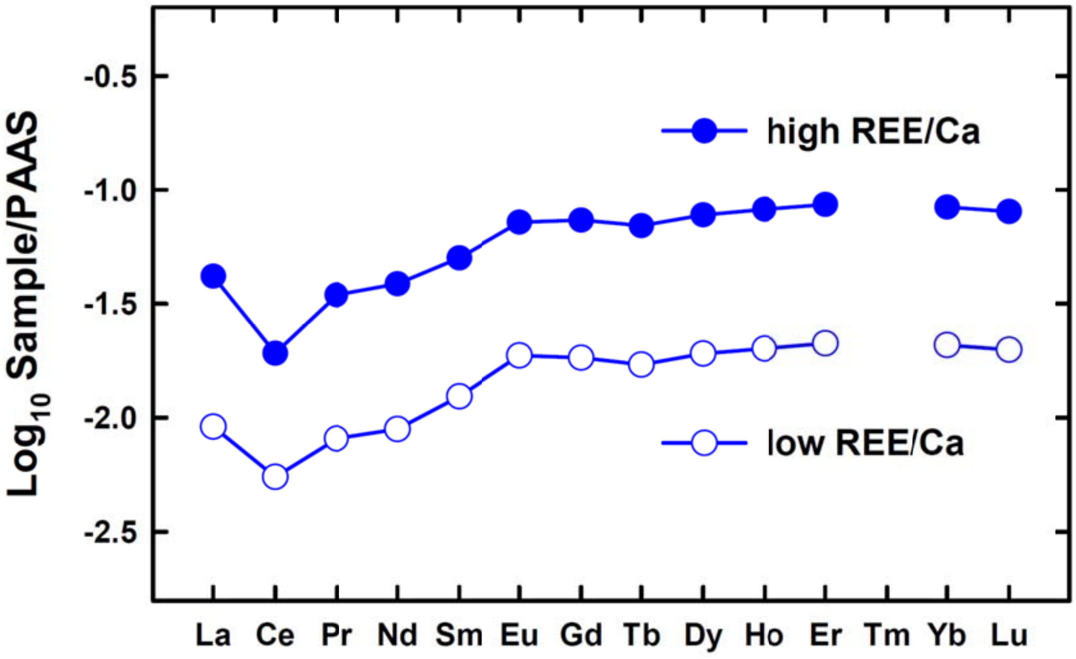

Supplementary Figure 5. Shale normalized (SN)[20] REE patterns. Averaged MD05-2925 *G. ruber* data with low REE/Ca ratios (Nd/Ca <0.25 µmol/mol) (hollow circles) and high REE/Ca ratios (Nd/Ca >0.75 µmol/mol) (solid circles).

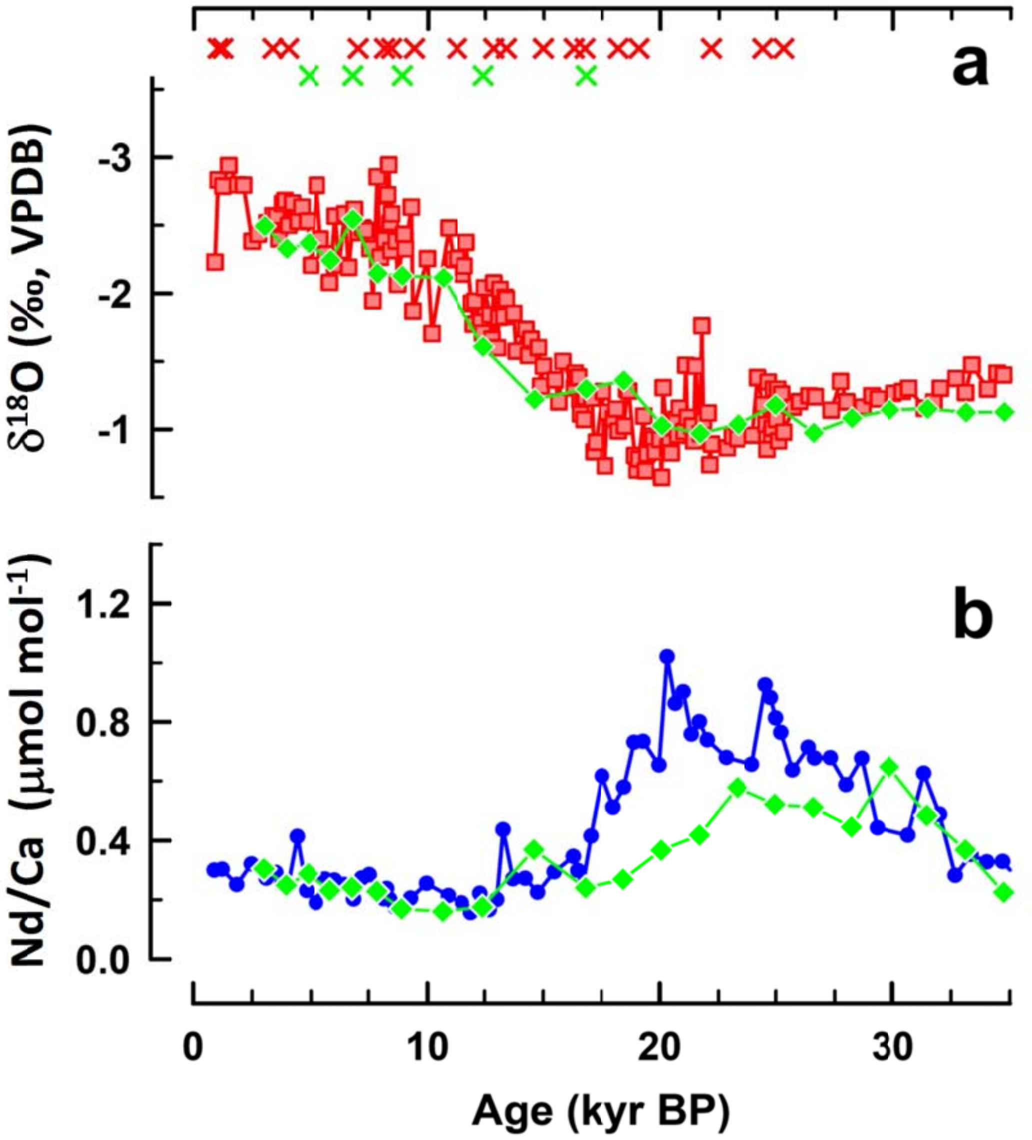

Supplementary Figure 6. Time series of foraminiferal $\delta^{18}O$ and Nd/Ca for MD05-2925 and ODP-1115B over the past 34 kyrs. (a) Planktonic foraminifera *G. ruber* $\delta^{18}O$ records of MD05-2925 (red) and ODP-1115B (green). Calibrated AMS ^{14}C dates are color-coded by core. Chronology of ODP-1115B was established using the ^{14}C dates and age control points at 57.8, 75.0, 112.0, 130.0, 135.0, and 167 kyr BP by matching the *G. ruber* $\delta^{18}O$ record with MD05-2925 sequence (Supplementary Fig. 12). **(b)** Co-variation of planktonic foraminifera *G. ruber* Nd/Ca records between MD05-2925 (blue) and ODP-1115B (green) over the past 34 kyr.

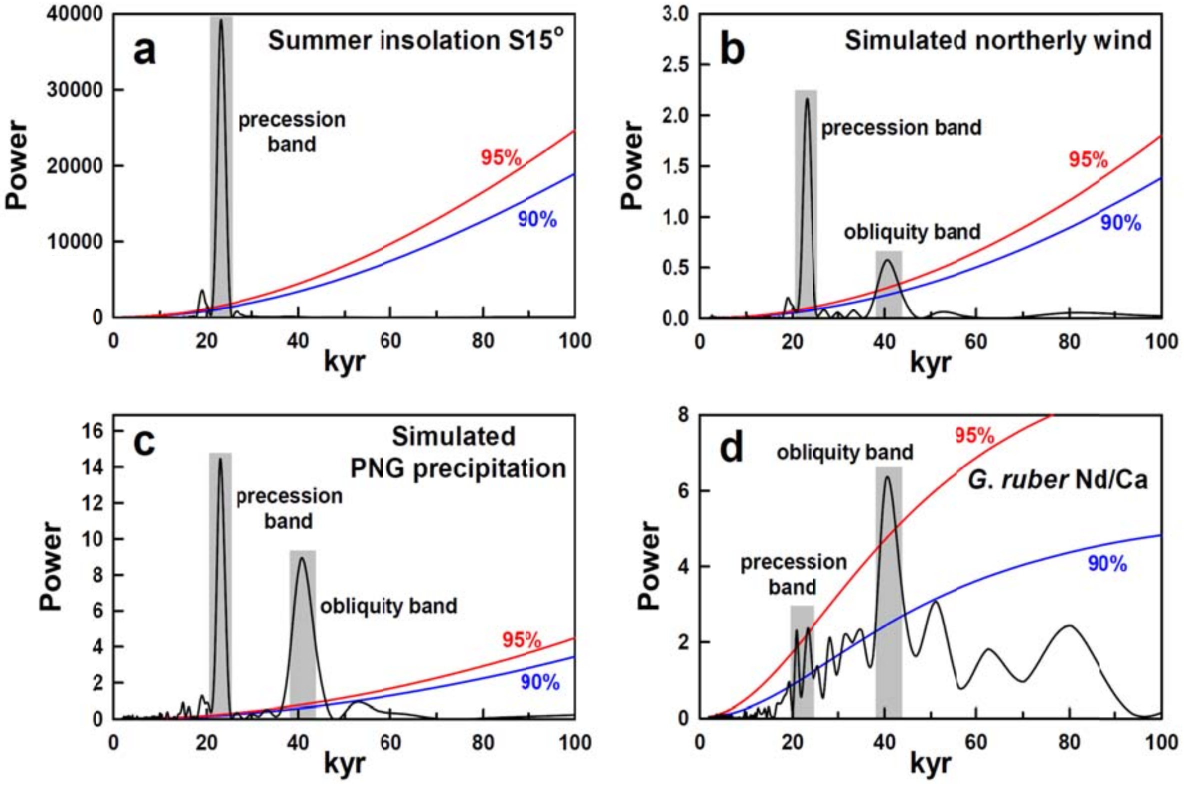

Supplementary Figure 7. Spectral power analysis results. **(a)** Southern hemisphere summer insolation (SHSI, 15 January) at 15°S[21] over the past 282 kyr, reflecting precession-dominated low-latitude insolation. **(b)** Simulated cross-equatorial 850 hPa northerly wind time series (0-20°S and 130-160°E), illustrated in Supplementary Figure 8b, with precession and obliquity bands. **(c)** Simulated PNG precipitation (5-12°S and 130-160°E). **(d)** MD 05-2925 planktonic foraminifera *G. ruber* Nd/Ca record. We used REDFIT v 3.8 (ref. 22) to perform spectral analyses. Red and blue lines respectively denote 95% and 90% significance levels of coherence. Vertical bars are the significant precession and obliquity bands.

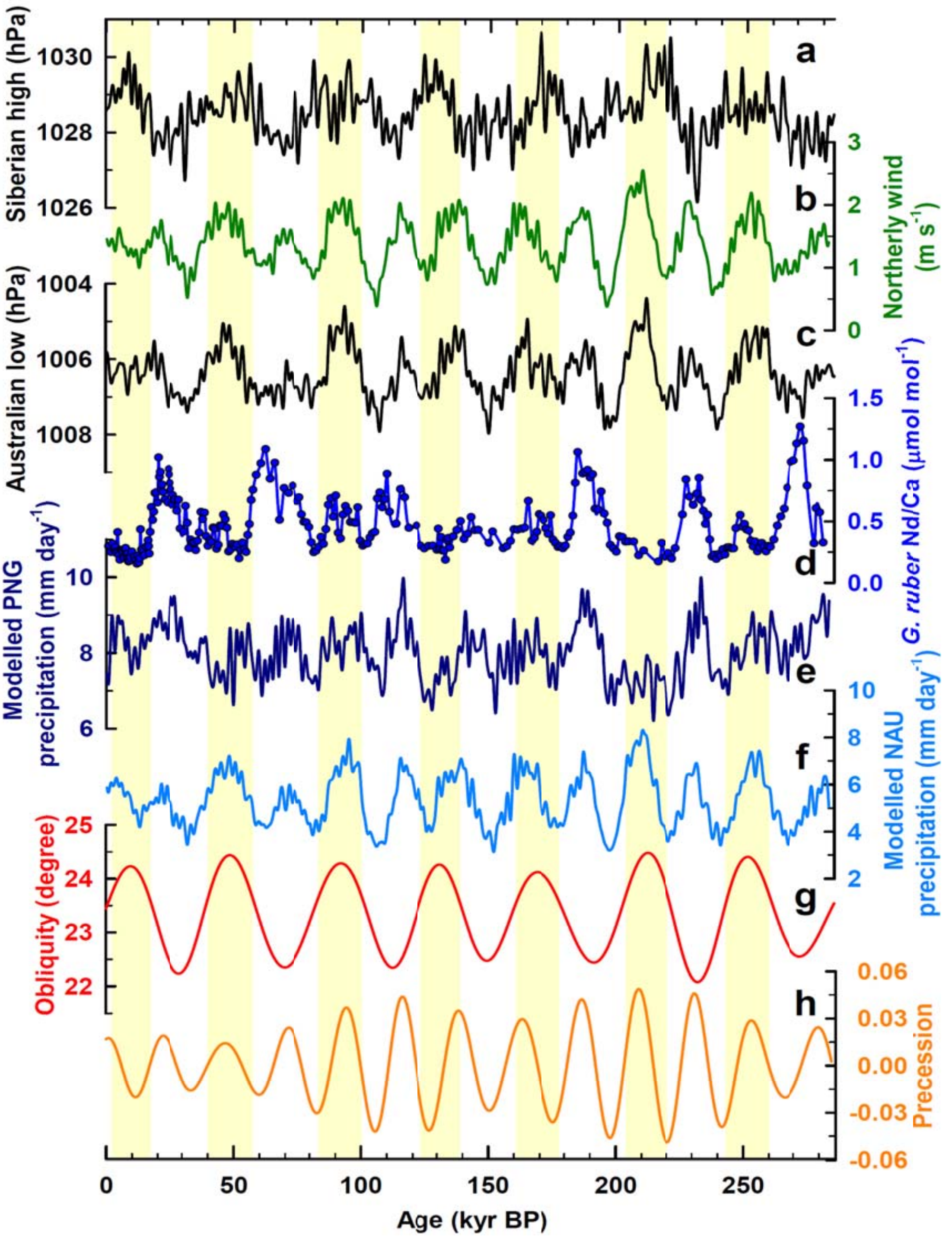

Supplementary Figure 8. Climatological results of the FOAM model simulation in January over the past 282 kyr. (a) Sea level pressure (SLP) for Siberian high (30-70°N and 60-130°E)[23,24]. **(b)** Cross-equatorial 850 hPa northerly wind (0-20°S and 130-160°E). **(c)** SLP for Australian low (10-35°S and 120-160°E)[23,24].**(e)** PNG precipitation (5-12°S and 130-160°E). **(f)** North Australia (NAU) precipitation (12-20°S and 120-160°E). **(d)** MD05-2925 *G. ruber* Nd/Ca record, **(g)** Earth obliquity, and **(h)** precession[21] are given for comparison. Vertical maize bars denote high-obliquity intervals.

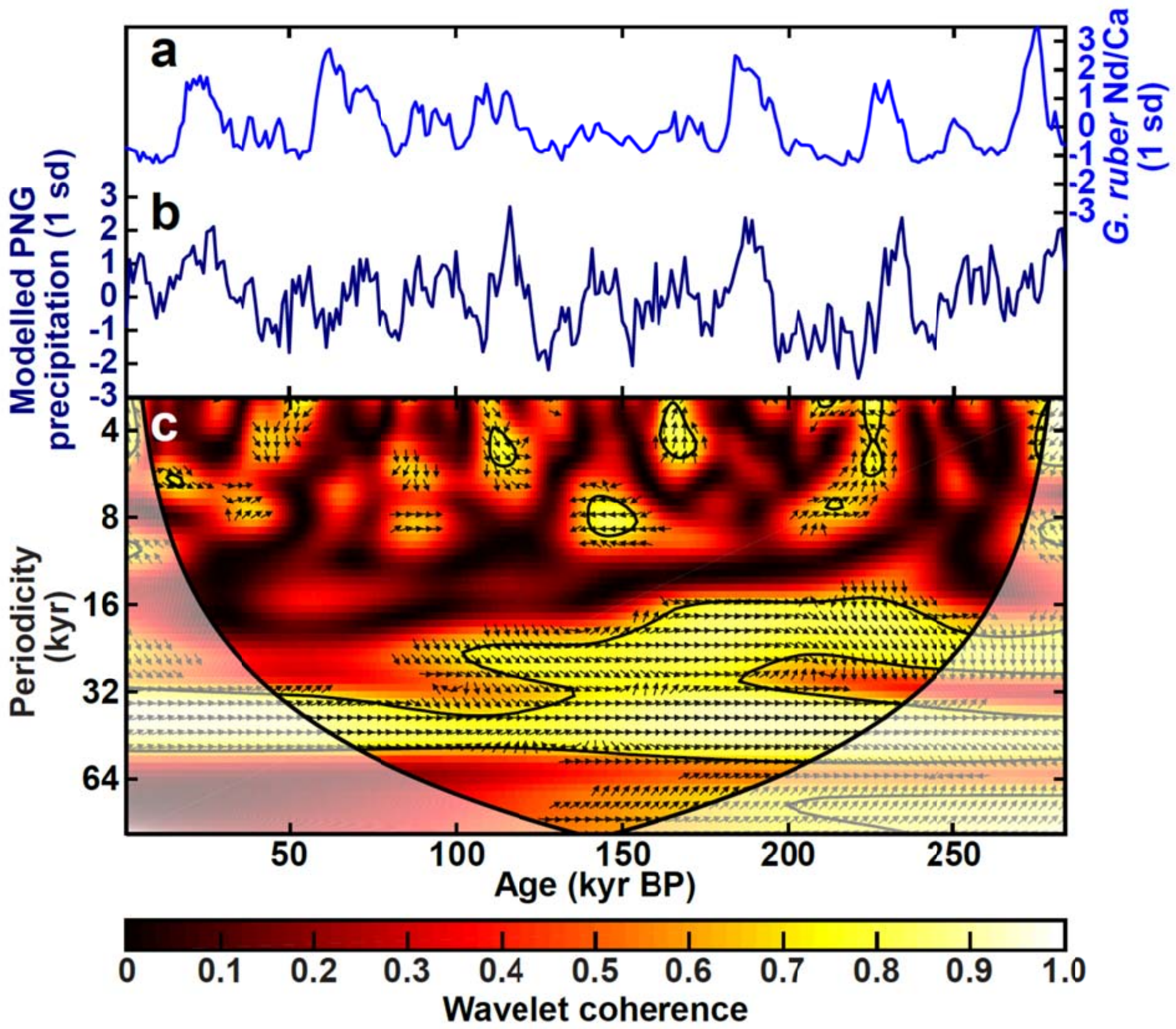

Supplementary Figure 9. Cross-wavelet coherence spectrum of **(a)** MD05-2925 *G. ruber* Nd/Ca with **(b)** modeled PNG precipitation, both standardized to one standard deviation (1 sd). **(c)** Coherence spectrum conducted by using Morlet mother wavelet[25,26] with thin black contour lines enclosing time-periodicity regions with significant concentrations of coherence (r^2) assessed by Monte Carlo simulation ($p = 0.05$)[26]. Heavy black line and shaded area is the cone of influence[25]. Arrows to the right indicate positive coherence. This spectral result shows the in-phase synchroneity of two records at both precession and obliquity periodicities.

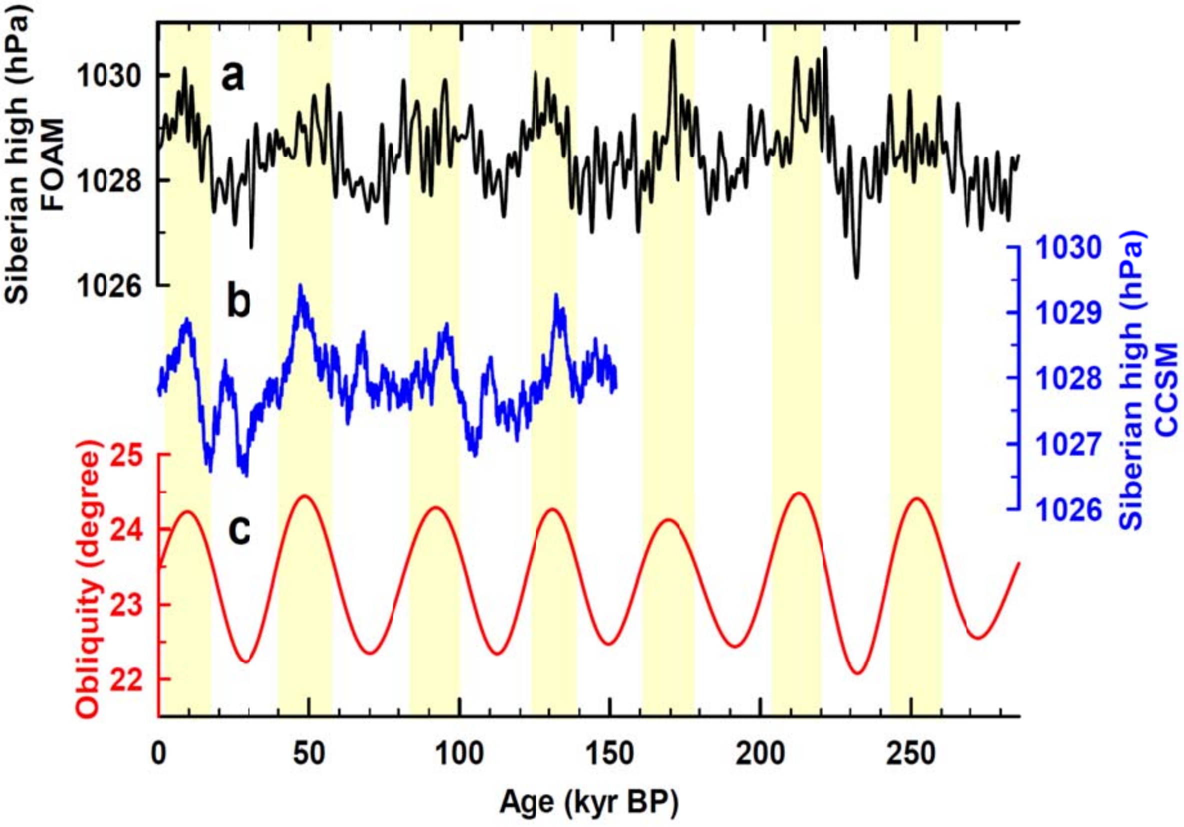

Supplementary Figure 10. Comparison between simulated sea level pressure (SLP) for Siberian high and Earth obliquity. Simulated SLP **(a)** over the past 282 kyr by FOAM[24] and **(b)** over the past 150 kyr by CCSM3[27]. **(c)** Earth obliquity[21]. Vertical maize bars denote high-obliquity intervals. Both simulations show high obliquity-induced strong high pressure cell.

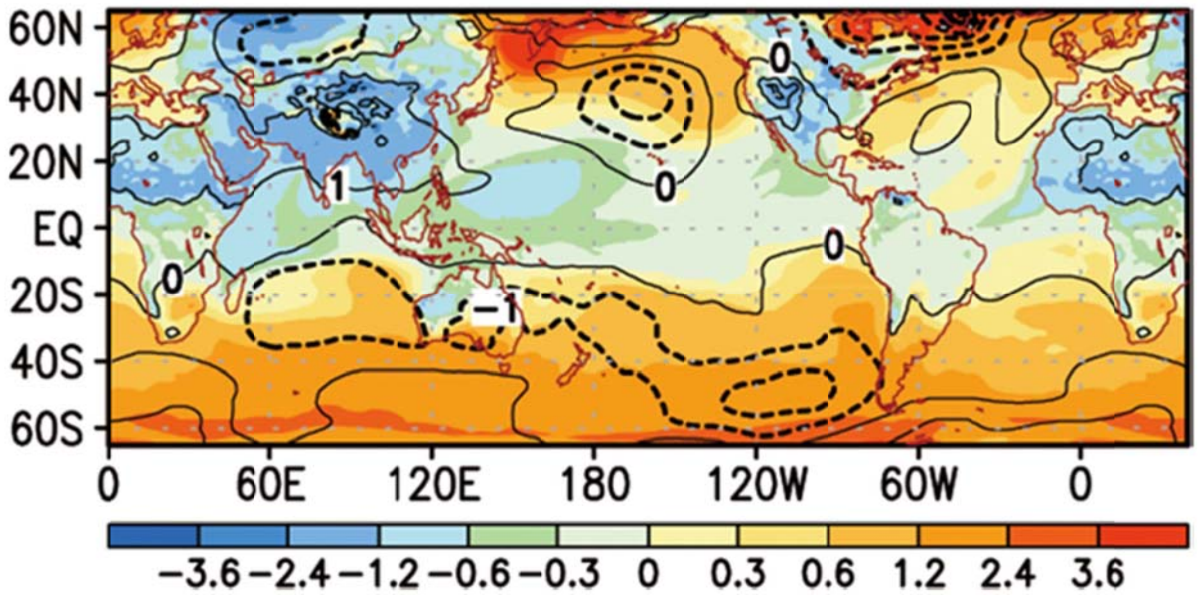

Supplementary Figure 11. Simulated DJF surface temperature (^oC in shading) and pressure (hPa in contour) differences between two experiments using high and low obliquity and similar low southern-hemisphere precessional phase (10 vs. 31 kyr BP). Both experiments were ran at 1-degree spatial resolution in slab ocean setting for 50 years using Community Earth System version 1 (CESM1)[28]**.** Experiments were forced only by obliquity and precessional changes with all other boundary conditions as of present day. Exact values of the respective parameters, eccentricity, obliquity, and precession index, used are 0.019605, 24.1552, and -0.015 at 10 kyr BP, and 0.015372, 22.4699, and -0.019 at 31 kyr BP. Under the similar precessional phase, the response can be treated as obliquity sensitivity and the simulated cold Siberian region and enhanced meridional pressure gradient over Asian continent during the northern hemisphere winter under the high-obliquity condition agrees with our observational data (Fig. 3) and proposing picture (Fig. 4). The model, CESM1 (http://www2.cesm.ucar.edu/), developed by the National Center for Atmospheric Research in Boulder, CO, sponsored by the National Science Foundation, USA, is one of the state-of-the-art coupled climate models that was used for Intergovernmental Panel on Climate Change (IPCC) future climate projection.

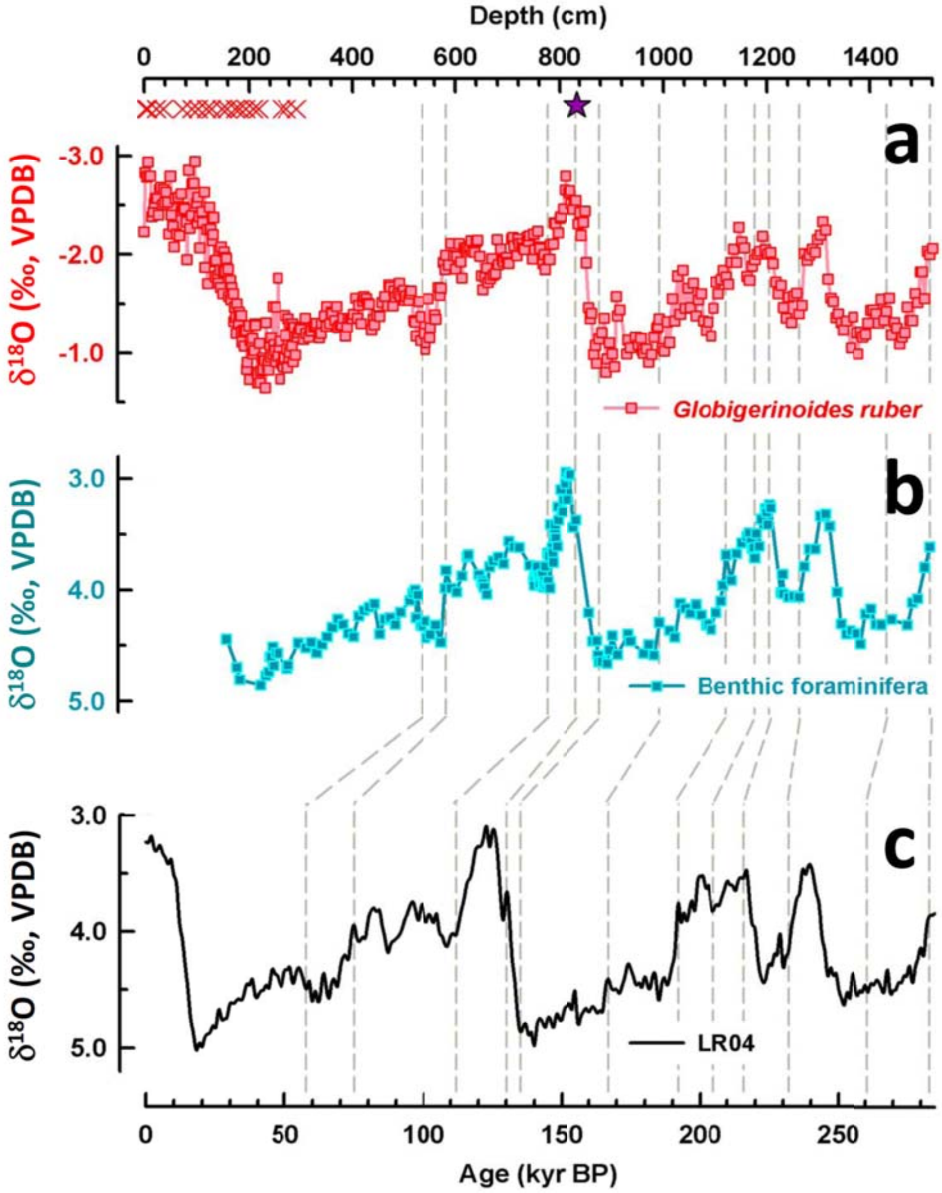

Supplementary Figure 12. Age model of core MD05-2925. (a) MD05-2925 *G. ruber* $\delta^{18}O$ record. Red cross symbols denote calibrated AMS ^{14}C dates used for upper 292 cm, and purple stars represent the last occurrence depth (830-835 cm) of *G. ruber* (pink). Dashed lines are the age control points by comparing **(b)** a composite MD05-2925 benthic foraminiferal $\delta^{18}O$ record with **(c)** global composite LR04 (ref. 29).

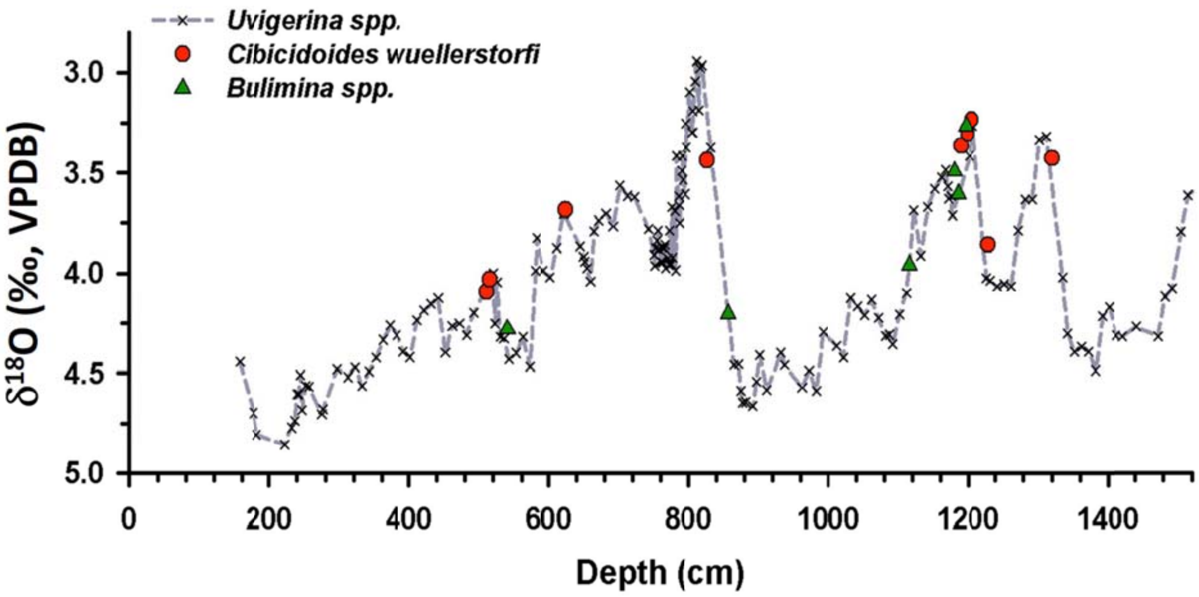

Supplementary Figure 13. Composite benthic foraminiferal oxygen isotope record of core MD05-2925. Cross symbols and a dark gray dashed line denotes the oxygen isotope data for *Uvigerina spp.* Red circles and green triangles are the corrected δ^{18}O data of *C. wuellerstorfi*[30] and *Bulimina spp.*[31], respectively.

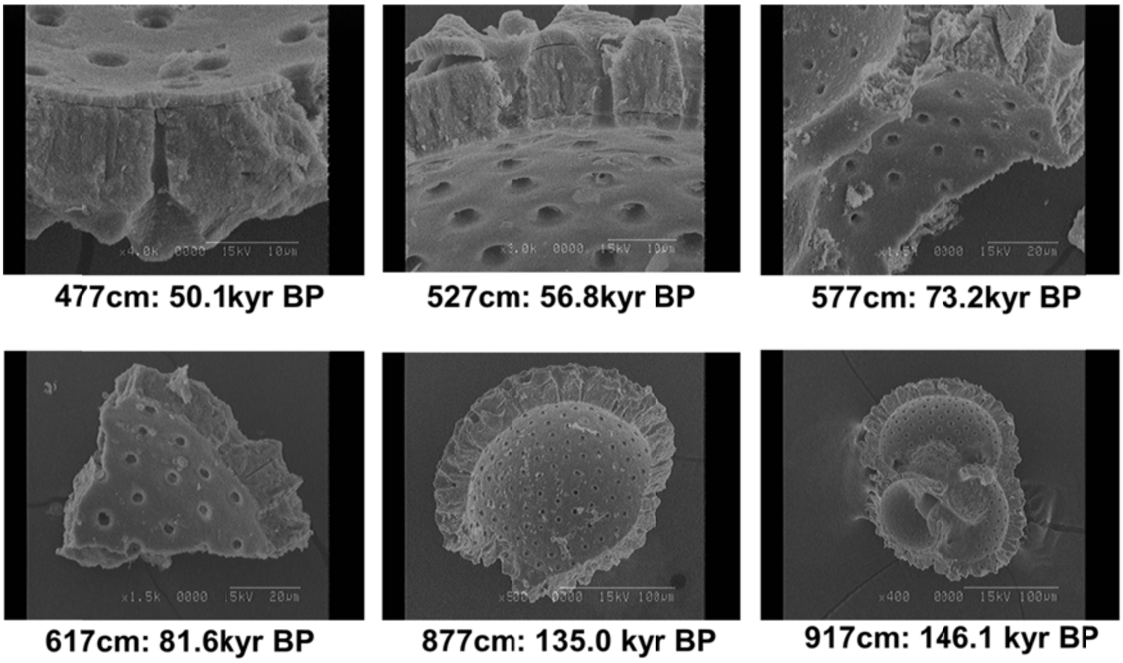

Supplementary Figure 14. Scanning electron microscope (SEM) images of foraminiferal shell of *G. ruber* from six depths. The foraminifera were washed with only ethanol and water before crushed for scanning. No post-depositional Mn-Fe nodules are observed.

Supplementary Table 1. AMS ^{14}C dates of core MD05-2925

Depth (cm)	Conventional ages ^{14}C age (yr) ± error		Calendar ages Dates ± error (2σ)		AMS Lab
**2**	1516	58	1023	141	U. Arizona[1]
**5**	1737	30	1223	79	GNS[2]
**22**	3534	41	3345	104	U. Arizona
**35**	4068	30	4013	123	GNS
**72**	6588	47	7027	138	U. Arizona
**90**	7751	35	8150	124	GNS
**102**	8030	65	8441	139	U. Arizona
**117**	8823	50	9414	111	GNS
**127**	10306	70	11259	159	U. Arizona
**147**	10411	70	12854	110	U. Arizona
**157**	12066	60	13392	84	GNS
**172**	13117	71	14973	309	U. Arizona
**180**	13748	35	16283	453	GNS
**192**	14080	74	16746	223	U. Arizona
**207**	15616	75	18201	175	GNS
**217**	16470	81	19083	90	U. Arizona
**262**	18985	94	22167	180	U. Arizona
**272**	20960	150	24411	167	U. Arizona
**292**	24650	78	25304	339	U. Arizona

[1] NSF-Arizona AMS Laboratory in University of Arizona (U. Arizona), Tucson, USA.

[2] Rafter Radiocarbon Laboratory, Institute of Geological and Nuclear Science (GNS), New Zealand.

Supplementary Table 2. Planktonic foraminifera and sediment Nd isotopic composition of core MD05-2925

Sample	Depth (cm)	Age (kyr BP)	Number of tests	$^{143}Nd/^{144}Nd$*	ε_{Nd}*
Foraminifera					
G. ruber	472-477	49.5-50.1	580	0.512584 ± 0.000016	-1.06 ± 0.32
G. ruber	537-542	58.8-60.6	250	0.512571 ± 0.000012	-1.30 ± 0.24
	472-473	49.5	-	0.512673 ± 0.000017	0.68 ± 0.32
Sediment	477-478	50.1	-	0.512642 ± 0.000020	0.08 ± 0.39
	537-538	58.8	-	0.512673 ± 0.000016	0.68 ± 0.31
	542-543	60.6	-	0.512655 ± 0.000013	0.34 ± 0.25

* Errors are 2σ of the mean.

Supplementary Note 1

Replicated foraminiferal REE/Ca records

To demonstrate the fidelity of MD05-2925 REE records, we performed a replication test on planktonic foraminifera *G. ruber* REE/Ca time series in the sedimentary core, ODP-1115B (9°11'S, 151°34'E; water depth 1149 m), from an adjacent site within the same climatic zone, 19 km north of the MD05-2925 site. For each 5-cm interval of the upper 486 cm of this core, 20-30 foraminiferal tests of down-core planktonic foraminifera *G. ruber* (white, *s.s.* 250-300 μm) were picked for measuring oxygen stable isotopes (Supplementary Fig. 6) to establish an age model. Trace elements over the past 34 kyr were analyzed (Supplementary Fig. 6). *G. ruber* REE/Ca variations in ODP-1115B are coherent with the MD05-2925 record (Supplementary Fig. 6). This synchroneity of two REE/Ca sequences demonstrates the robustness of our methodology and interpretations.

Evaluation of different factors on foraminiferal REE contents

Serious influences of metallic oxides on foraminiferal REE contents were reported for the marine sedimentary cores and traps, especially in the Atlantic[1-3]. Under an oxidative condition, Fe/Ca ratios in our samples (<50 μmol mol^{-1}) are much lower than the reported values (>100 μmol mol^{-1}) for the diagenetically contaminated foraminiferal test from anoxic sites[4]. Previous studies showed that REE and Fe are positively correlated due to authigenic coatings[1,3,5]. An absence of a relationship between Nd/Ca and Fe/Ca (Supplementary Fig. 3b) also suggests that foraminiferal test REE/Ca variation is not attributed to this coating phase.

The average Nd concentration of towed open-ocean planktonic foraminifera is ~0.1 ppm[6], which is free from diagenetic contamination. Nd levels in foraminiferal calcite lattice at our study site with massive inputs of terrestrial materials should be larger than the average value. The low Nd content of our foraminifera is ~0.2 ppm. Using the Nd value of modern PNG coastal surface seawater (8.35 pmol kg^{-1})[7] and K_d values of 200–500 (refs. 4), the Nd/Ca in foraminiferal calcite is expected to be about 0.16-0.41 μmol mol^{-1}, which is consistent with the core-top foraminifera *G. ruber* values of 0.19-0.41 μmol mol^{-1} (Supplementary Fig. 2). A lower Kd values of 16-52 (ref. 6) and 4-302 (ref. 8) were also reported by other studies. These discrepancies could be caused by that seawater and foraminifera samples were not from the same sampling position[6,8]. However, using the high end range of these lower Kd, our data are still consistent with the predicted range of Nd levels.

Foraminiferal test ε_{Nd} has been used to reconstruct seawater neodymium isotopic composition in the past[6,9,10]. However, the source of ε_{Nd} in sedimentary foraminifera is debated. Sedimentary planktonic foraminiferal Nd isotopes have been argued to record the bottom water Nd isotopic composition, testifying to the formation of authigenic coatings in bottom water or pore water, even in samples that have been subjected to chemical cleaning[11]. Our ε_{Nd} data tend to support that a surface water signal was recorded in the cleaned test in this study. The radiogenic ε_{Nd} values of planktonic foraminifera *G. ruber* are -1.06 ± 0.32 at ~50 kyr BP with low REE levels and -1.30 ± 0.24 at ~60 kyr BP with high REE contents (Supplementary Fig. 2). The ε_{Nd} values are different from 0.1~0.7 for the surrounding sediment (Supplementary Table 2) and -4 for

the seawater below 600 m depths near the Solomon Sea (stations of EUC-Fe 30, FLUSEC 43 and FLUSEC 22 in the ref. 7). Although the sites in the Solomon Sea are ~500 km away from our site, the ε_{Nd} value (-4) can show the local deep seawater compositions at our site and be used for comparison. The reason is that the deep western boundary current always flows northwestward along the east coast of PNG[7] and our site is located at the current path among stations of EUC-Fe 30, FLUSEC 43 and FLUSEC 22. REE concentration and Nd isotope data of planktonic foraminifera tests from plankton tows and sediment traps in the NW Atlantic show 80% of Nd is associated with authigenic metal oxides and organic matter, which form in the water column[3]. In our study area, the planktonic foraminiferal Nd isotope data, however, do not reflect those of deep to bottom waters. The evidence also suggests that the foraminiferal test REE source is not from deep/bottom seawater or sediment by post-depositional diagenesis.

If planktonic foraminiferal REE of MD05-2925 were from different surface water masses associated with seasonal ocean circulation, different ε_{Nd} values would be expected on the basis of wide range of +2 to -2 for modern surface water ε_{Nd} values in the equatorial Pacific[7]. Indistinguishable ε_{Nd} values (Supplementary Fig. 2, Supplementary Table 2) at two different ages of 49.5-50.1 kyr BP with low foraminiferal Nd levels of 0.30-0.32 μmol mol^{-1} and 58.8-60.6 kyr BP with high foraminiferal Nd contents of 0.88-0.97 μmol mol^{-1} suggest a single dominant source.

Could boundary exchange[12], a mechanism involving the release of dissolved REEs to seawater from particulates in the marine shelf setting, result in planktonic

foraminifera *G. ruber* REE variability? It is unlikely given the difference in isotopic composition between the regional seawater and local lithologies with very radiogenic Nd[7,12]. Neither is there any reason to expect that boundary exchange could change significantly over the past 282 kyr, as our REE patterns (Supplementary Fig. 5) and ε_{Nd} data (Supplementary Fig. 2) in the foraminifera do not show any change.

The Australian continent is the largest aeolian dust source in this region. However, the aeolian dust is not expected to be transported to our study site[13]. Dust deposition from the remote East Asian continent is also negligible[13]. Besides, the shale-normalized flat REE pattern of aeolian dust[14] is distinctively different from the planktonic foraminiferal pattern (Supplementary Fig. 5).

A noticeable in-phase correlation between Nd/Ca and Mn/Ca variations is observed in Supplementary Figures 3c and 4. This relationship could conceivably imply redox-induced micron-scale precipitation of $MnCO_3$ (ref. 3). High Mn and REE concentrations in planktonic foraminifera could be caused by oxide dissolution under reducing conditions and re-mobilization of redox sensitive ions[3]. These diagenetic source of Mn and REE are accompanied by positive Ce anomalies [Ce* = $(3Ce_{SN})/(2La_{SN} + Nd_{SN})$]. However, there is no clear correlation (R^2 = 0.069) between records of Mn/Ca and Ce anomalies (Supplementary Fig. 4b, 4c) and the Ce anomalies is always negative. This is also supported by the lack of correlation between Mn/Ca and Fe/Ca. The close correlation of Mn and Fe is a common feature of diagenetic source or authigenic coatings[3,5]. These features suggest that such a reaction is not manifest in our REE/Ca records. Planktonic foraminiferal Mn/Ca ratios have been considered as a tracer

for dissolved terrestrial input[15]. In-phase variations of Mn/Ca and REE/Ca are here attributed to common terrestrial sources from PNG.

Supplementary References

1. Palmer, M. R. Rare earth elements in foraminifera tests, *Earth Planet. Sci. Lett.* **73**, 285-298 (1985).
2. Roberts, N. L., Piotrowski, A. M., McManus, J. F. & Keigwin, L. D. Synchronous deglacial overturning and water mass source changes. *Science* **327**, 75-78 (2010).
3. Roberts, N. L., Piotrowski, A. M., Elderfield, H., Eglinton, T. I. & Lomas, M. W. Rare earth element association with foraminifera. *Geochim. Cosmochim. Acta* **94,** 57-71 (2012).
4. Haley, B. A., Klinkhammer, G. P. & Mix, A. C. Revisiting the rare earth elements in foraminiferal tests. *Earth Planet. Sci. Lett.* **239,** 79-97 (2005).
5. Tachikawa, K., Toyofuku, T., Basile-Doelsch, I. & Delhaye, T. Microscale neodymium distribution in sedimentary planktonic foraminiferal tests and associated mineral phases. *Geochim. Cosmochim. Acta* **100,** 11-23 (2013).
6. Pomiès, C. Davies, G. R. & Conan, S. M.-H. Neodymium in modern foraminifera from the Indian Ocean: implications for the use of foraminiferal Nd isotope compositions in paleooceanography. *Earth Planet. Sci. Lett.* **203,** 1031-1045 (2002).
7. Grenier, M. *et al.* From the subtropics to the central equatorial Pacific Ocean: Neodymium isotopic composition and rare earth element concentration variations. *J. Geophys. Res. -Oceans* **118**, doi:10.1029/2012JC008239 (2013).
8. Martinez-Boti, M. A., Vance, D., & Mortyn, P. G. Nd/Ca ratios in plankton-towed and core top foraminifera: Confirmation of the water column acquisition of Nd. *Geochem. Geophys. Geosyst.* **10**, doi: 10.1029/2009GC002701 (2009).
9. Burton, K. W. & Vance, D. Glacial-interglacial variations in the neodymium isotope composition of seawater in the Bay of Bengal recorded by planktonic foraminifera. *Earth Planet. Sci. Lett.* **176,** 425-441 (2000).
10. Stoll, H. M., Vance, D. & Arevalos, A. Records of the Nd isotope composition of seawater from the Bay of Bengal: Implications for the impact of Northern Hemisphere cooling on ITCZ movement. *Earth Planet. Sci. Lett.* **255,** 213-228 (2007).
11. Tachikawa, K., Piotrowski, A. M. & Bayon, G. Neodymium associated with foraminiferal carbonate as a recorder of seawater isotopic signatures. *Quat. Sci. Rev.* **88**, 1-13 (2014).
12. Lacan, F. & Jeandel, C. Tracing Papua New Guinea imprint on the central Equatorial Pacific Ocean using neodymium isotopic compositions and Rare Earth Element patterns. *Earth Planet. Sci. Lett.* **186,** 497-512 (2001).
13. Jickells, T. D. *et al.* Global iron connections between desert dust, ocean biogeochemistry, and climate. *Science* **308,** 67-71 (2005).
14. Gabrielli, P. *et al.* Ultra-low rare earth element content in accreted ice from sub-glacial Lake Vostok, Antarctica. *Geochim. Cosmochima. Acta* **73**, 5959-5974 (2009).

15. Klinkhammer, G. P., Mix, A. C. & Haley, B. A. Increased dissolved terrestrial input to the coastal ocean during the last deglaciation. *Geochem. Geophys. Geosys.* **10,** Q03009, doi: 10.1029/2008GC002219 (2009).
16. Fallon, S. J., White, J. C. & McCulloch. M. Porites corals as recorders of mining and environmental impacts: Misima Island, Papua New Guinea. *Geochim. Cosmochima. Acta* **66**, 45-62 (2002).
17. Zhang, J. & Nozaki, Y. Rare earth elements and yttrium in seawater: ICP-MS determinations in the East Caroline, Coral Sea, and South Fiji basins of the western South Pacific Ocean. *Geochim. Cosmochim. Acta* **60**, 4631-4644 (1996).
18. Wyndham, T., McCulloch, M., Fallon, S. & Alibert, C. High-resolution coral records of rare earth elements in coastal seawater: biogeochemical cycling and a new environmental proxy. *Geochim. Cosmochim. Acta* **68,** 2067-2080 (2004).
19. Fitzsimmons, K. E., Miller, G. H., Spooner, N. A. & Magee, J. W. Aridity in the monsoon zone as indicated by desert dune formation in the Gregory Lakes basin, northwestern Australia. *Aust. J. Earth Sci.* **59,** 469-478 (2012).
20. McLennan, S. M. Rare earth elements in sedimentary rocks; influence of provenance and sedimentary processes. *Rev. Mineral. Geochem.* **21,** 169-200 (1989).
21. Berger, A. L. Long-term variations of caloric insolation resulting from the Earth's orbital elements. *Quat. Res.* **9,** 139-167 (1978).
22. Schulz, M. & Mudelsee, M. REDFIT: Estimating red-noise spectra directly from unevenly spaced paleoclimatic time series. *Comput. Geosci.* **28**, 421-426 (2002).
23. Kutzbach, J. E., Liu, X., Liu, Z. & Chen, G. Simulation of the evolutionary response of global summer monsoons to orbital forcing over the past 280,000 years. *Clim. Dynam.* **30,** 567-579 (2008).
24. Shi, Z. *et al.* Distinct responses of East Asian summer and winter monsoons to astronomical forcing. *Clim. Past* **7,** 1363-1370 (2011).
25. Torrence, C. & Compo, G. P. A practical guide to wavelet analysis, *Bull. Am. Meteorol. Soc.*, **79**, 61-78 (1998).
26. Grinsted, A., Moore, J. C. & Jevrejeva, S. Application of the cross wavelet transform and wavelet coherence to geophysical time series. *Nonlin. Processes Geophys.* **11**, 561-566 (2004).
27. Li, X., Liu, X., Qiu, L., An, Z. & Yin, Z. Transient simulation of orbital-scale precipitation variation in monsoonal East Asia and arid central Asia during the last 150 ka. *J. Geophys. Res. Atmos.* **118**, 7481-7488, doi:10.1002/jgrd.50611 (2013).
28. Hurrell, J. W. *et al.* The Community Earth System Model: A framework for collaborative research. *Bull. Amer. Meteor. Soc.* **94**, 1339-1360, doi:10.1175/BAMS-D-12-00121.1 (2013).
29. Lisiecki, L. E. & Raymo, M. E. Pliocene-Pleistocene stack of 57 globally distributed benthic $\delta^{18}O$ records. *Paleoceanography* **20,** PA1003, doi: 10.1029/2004PA001071 (2005).
30. Shackleton, N. J. & Opdyke, N. D. Oxygen isotope and palaeomagnetic stratigraphy of Equatorial Pacific core V28-238: Oxygen isotope temperatures and ice volumes on a 10^5 year and 10^6 year scale. *Quat. Res.* **3**, 39-55 (1973).

31. Oba, T. *et al.* Paleoceanographic change off central Japan since the last 144,000 years based on high-resolution oxygen and carbon isotope records. *Global Planet. Change* **53**, 5-20 (2006).
